# Correlation‐Driven *d*‐Band Modifications Promote Chemical Bonding at 3*d*‐Ferromagnetic Surfaces

**DOI:** 10.1002/smll.202508952

**Published:** 2025-12-03

**Authors:** David Maximilian Janas, Andreas Windischbacher, Alessandro Sala, Vitaliy Feyer, Iulia Cojocariu, Manuel Gruber, Mehdi Bouatou, Andrea Droghetti, Peter Puschnig, Giovanni Zamborlini, Mirko Cinchetti

**Affiliations:** ^1^ Department of Physics TU Dortmund University 44227 Dortmund Germany; ^2^ Institute of Physics NAWI Graz, University of Graz Universitätsplatz 5 Graz 8010 Austria; ^3^ CNR – Istituto Officina dei Materiali (IOM) S.S. 14 km 163.5, Area Science Park, Basovizza Trieste 34149 Italy; ^4^ Peter Grünberg Institute (PGI‐6) Forschungszentrum Jülich GmbH 52425 Jülich Germany; ^5^ Faculty of Physics and CENIDE University of Duisburg–Essen 47057 Duisburg Germany; ^6^ Department of Molecular Sciences and Nanosystems Ca’ Foscari University of Venice via Torino 155 Mestre‐Venice 30170 Italy

**Keywords:** chemisorption, d‐band model, electron correlation, ferromagnetic surface, metal/organic interface

## Abstract

Understanding chemical bonding at molecule–metal interfaces is essential for advancing applications in catalysis, spintronics, and organic electronics. While the Newns–Anderson and *d*‐band models have provided key insights, their applicability remains limited in systems involving large organic adsorbates and correlated metallic substrates. This work investigates the interaction between pentacene (5A) and an oxygen‐passivated Fe(100) surface (Fe–O), where oxygen chemisorption gives rise to strong electronic correlations. A combination of photoemission orbital tomography, scanning tunneling spectroscopy, and electronic structure calculations reveals pronounced hybridization between 5A frontier orbitals and the Fe *d*‐states. A tailored DFT+U approach with a negative effective on‐site interaction (*U*_eff_ = −3.1 eV) captures the experimentally observed reduction in *d*‐band spin splitting and narrowing, consistent with dynamical mean‐field theory. These correlation‐induced modifications enhance the energetic overlap between metal *d*‐states and molecular orbitals, driving a transition from physisorption to strong chemisorption. Building on these insights, the *d*‐band model is extended to include spatially modulated adsorbate–substrate coupling, successfully reproducing the experimentally observed orbital substructures. These findings offer a tractable route for incorporating many‐body effects into simplified chemisorption models, enabling predictive insights into molecule–metal bonding at correlated surfaces and guiding the design of 3*d*‐metal catalysts and organic spintronic interfaces.

## Graphical abstract

Electron correlation at the oxygen‐passivated Fe(100) surface renormalizes and narrows the Fe *d*‐bands, enhancing their energetic overlap with the frontier orbitals of adsorbed pentacene. This strengthens hybridization and converts a weakly bound interface into a chemisorbed hybrid system, illustrating how correlation‐tuned *d*‐bands can be used to control molecule–metal bonding and interfacial reactivity at catalytic and spintronic interfaces.

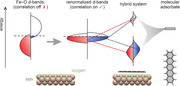

## 1 Introduction

### 1.1 Simplified Models to Treat the Adsorbate–Metal Problem

Developing theoretical models that rationalize the chemical bonding at adsorbate–metal interfaces is of utmost importance in various fields, ranging from organic opto‐electronics, to spintronics and catalysis. On the one hand, the nature of molecule–metal interaction dictates the efficiency of charge and spin injection across the interface, which can be harnessed in devices for energy harvesting and data storage.^[^ ^1^, ^2^ ^]^ On the other hand, the emerging bonds determine the chemisorption energies of adsorbates in electrocatalytic conversion processes, which are essential, for example, for the production of green fuels and the synthesis of ammonia.^[^ ^3^, ^4^ ^]^

Our fundamental understanding of these interfaces relies primarily on simplified, single‐particle‐based chemisorption models, which make use of straightforward descriptors to predict the chemical bond formation at surfaces and interfaces.^[^ ^5^, ^6^ ^]^ The Newns–Anderson model, introduced in 1969,^[^ ^7^, ^8^ ^]^ was the first theoretical framework to provide a microscopic description of the interaction between adsorbates and metal surfaces, whose basic concepts remain highly relevant to this day. This model demonstrated that key characteristics of adsorbate–metal systems, such as their chemisorption energies, could be derived from two fundamental properties: 1) the density of states (DOS) of the metal, and 2) the spatial overlap between the metal and adsorbate states wavefunctions.

Hammer and Nørskov later refined these principles by separating the interaction of the adsorbate with the metal into contributions of the metal's broad *sp*‐bands and its narrow *d*‐bands, leading to the development of the widely used *d*‐band model of chemisorption.^[^ ^6^, ^9^ ^]^ A key achievement of the *d*‐band model was the identification of the center of the metal *d*‐bands as an effective descriptor for predicting the reactivity of metal surfaces. More specifically, the model describes how the interaction between localized metal *d*‐states and adsorbate states leads to the formation of bonding and antibonding states with mixed metal–molecule character.^[^ ^6^ ^]^ In ferromagnetic (FM) metals, this bond formation is furthermore spin‐dependent, and therefore closely tied to the magnetic properties of the substrate.^[^ ^10^, ^11^ ^]^

The *d*‐band model has been instrumental in elucidating reactivity trends across the periodic table^[^ ^12^ ^]^ and in understanding the effects of alloying^[^ ^13^, ^14^ ^]^ or strain^[^ ^15^ ^]^ on surface reactivity. More recently, it has also been used to predict and explain a rise in catalytic activity of FM transition metal (TM) surfaces upon quenching their surface magnetic properties.^[^ ^16^, ^17^ ^]^ Its ability to simplify complex interactions has made it a cornerstone in the fields of surface science and catalysis. Nonetheless, some aspects of the model remain largely unexplored. In particular, while both the Newns–Anderson and the *d*‐band model have been extensively used to describe the interaction between metal surfaces and small adsorbates consisting of only a few atoms, their applicability to quantify the surface hybridization of larger organic adsorbates is more nebulous.^[^ ^18^, ^19^ ^]^ Moreover, these models are typically applied using parameters derived from the Kohn–Sham states of density functional theory (DFT) within standard local or semi‐local approximations, which do not account for modifications to the *d*‐band structure arising from many‐body effects beyond a static mean‐field (Stoner‐like) description. These effects can be significant at FM TM surfaces and dominate their magnetic properties.^[^ ^20^, ^21^, ^22^, ^23^ ^]^ Extending the models in both directions, toward larger adsorbates and toward correlated substrates, is crucial for advancing the understanding of more complex adsorbate–surface interactions. Beyond this conceptual interest, a correlation‐aware description of chemisorption at ferromagnetic metal surfaces would also enable more rational tuning of adsorption energies and spin‐dependent hybridization, with direct implications for optimizing catalytic activity and controlling charge and spin transport in organic–metal hybrid devices.

Here, we take on this double challenge by studying pentacene (5A) molecules adsorbed on an oxygen‐passivated Fe surface (Fe–O) as a model system. While 5A is a simple, yet large polycyclic aromatic hydrocarbon widely used in organic electronics,^[^ ^24^, ^25^, ^26^, ^27^ ^]^ the Fe–O surface is known for its enhanced electron correlation triggered by the oxygen (O) chemisorption.^[^ ^23^ ^]^ Photoemission orbital tomography (POT), scanning tunneling microscopy and spectroscopy (STM and STS), combined with electronic structure calculations, reveal the formation of substantial molecule–metal bonds at the interface. Importantly, our calculations show that incorporating an effective correction within DFT, which emulates the correlation‐induced renormalization of Fe *d*‐band spin splitting observed in dynamical mean‐field theory (DMFT),^[^ ^23^ ^]^ is essential for interpreting the experimental data. Building on these insights, we propose an adapted *d*‐band framework that: (1) accounts for the renormalization of the single‐particle *d*‐band structure due to electron correlations, and (2) captures the laterally inhomogeneous interactions between substrate *d*‐states and the molecular orbitals (MOs) of extended adsorbates.

## 2 Results and Discussion

### 2.1 Correlation‐Induced *d*‐Band Modifications

To understand the role of electron correlation effects in chemisorption, it is essential to first examine the electronic properties of the Fe(100) surface passivated with oxygen, which forms the Fe(100)–*p*(1 × 1)O reconstruction (Fe–O).^[^ ^28^ ^]^ In our previous work,^[^ ^23^ ^]^ we observed a significant reduction in the magnetic moment of surface Fe atoms upon oxygen passivation — a result not predicted by conventional DFT.

**Figure 1:**
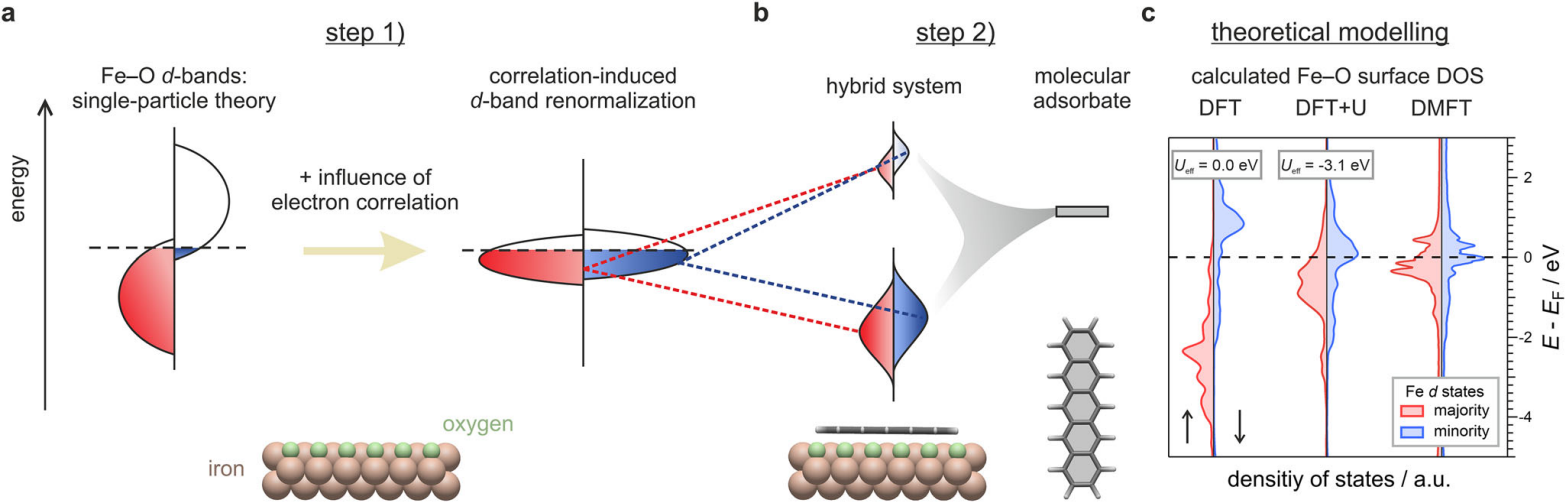
How electron correlations affect the predictions of the d‐band theory of chemisorption. a) Left: Sketch of the spin‐resolved d‐PDOS of the Fe–O surface within the single‐electron framework. Right: Renormalization of the spin‐resolved d‐PDOS of Fe–O due to enhanced electron correlation. A model of the Fe–O surface is displayed at the bottom, where O and Fe atoms are displayed as green and brown spheres, respectively. b) Schematic representation of the interaction between a molecular state and the renormalized d‐bands of the Fe–O surface. c) Juxtaposition of calculated d‐PDOS of the Fe–O surface resulting from DFT, DFT+U and DMFT. The case of U_eff_ = 0 eV corresponds to standard DFT calculations. For the DFT+U calculations a value of U_eff_ = U − J = −3.1 eV was employed.

DFT typically describes the *d*‐bands within an effective single‐particle framework, approximated by the Kohn–Sham states and treated within a Stoner‐like picture of magnetism.^[^ ^29^ ^]^ In this picture, bond formation with oxygen results in only minor modifications to the electronic structure. For example, DFT calculations using the generalized gradient approximation (GGA) predict a clear spin splitting of the Fe *d*‐states, with the majority‐spin band (red) lying below the Fermi energy *E_F_* and the minority‐spin band (blue) mostly above it — similar to the behavior of a clean Fe surface (**Figure** **1**a, left panel; Figure 1c, leftmost panel).

In general, when electron correlation is taken into account, the electronic structure of transition‐metal surfaces can undergo substantial modifications. In the case of pristine Fe(100), however, correlation remains relatively weak, leading only to a partial renormalization of the DFT‐predicted band structure that still exhibits a Stoner‐like exchange splitting.^[^ ^30^ ^]^ In contrast, adsorbate‐induced changes in the *d*‐shell occupancy arising from charge transfer can enhance electronic correlations and thereby profoundly alter the substrate's electronic structure. For oxygen chemisorption, this enhancement causes a breakdown of the Stoner picture of ferromagnetism,^[^ ^23^ ^]^ marked by an unexpected reduction of the surface exchange splitting and a narrowing of the *d*‐band width (Figure 1a, right), as well as the emergence of satellite features several eV below *E*_F_. All these changes originate from many‐body scattering processes, which lie beyond the capabilities of static mean‐field methods like DFT, but are accurately captured by combining DFT with DMFT.^[^ ^31^, ^32^ ^]^ The latter incorporates a frequency‐dependent complex self‐energy and thus provides a more complete picture of correlation effects in ferromagnetic systems.^[^ ^22^, ^23^ ^]^

While DMFT provides an accurate description of transition metals, its computational cost makes it impractical for large‐scale modelling of complex hybrid interfaces such as the organic/Fe–O system studied here. A more computationally tractable approach is therefore needed — one that can mimic key correlation‐induced features of the electronic structure via an *ad hoc* and “computationally light” correction to the DFT band structure.

To this end, we utilize the DFT+U method, introducing a *negative* effective on‐site parameter *U*_eff_ *= U – J* to phenomenologically modify the Fe *d*‐bands. In its conventional formulation, DFT+U^[^ ^33^, ^34^ ^]^ is a static mean‐field approach, applied with a positive *U*_eff_ to represent a screened Coulomb repulsion. In surface science, it has been used to correct local magnetic moments in coordinated metal ions of molecular adsorbates (*e.g*., metal‐phthalocyanines)^[^ ^35^, ^36^, ^37^ ^]^ or to improve bandgaps in semiconducting substrates.^[^ ^38^, ^39^ ^]^ However, for metallic Fe surfaces, conventional DFT+U with positive *U*_eff_ applied to the *d*‐bands tends to increase spin splitting, thereby worsening agreement with experiment — a limitation recognized since the method's early applications.^[^ ^33^ ^]^ For such systems, the inclusion of dynamical correlations via a self‐energy becomes essential to renormalize the DFT+U predictions with a positive *U*_eff_.^[^ ^30^ ^]^

In contrast to this conventional usage and its limitations, our choice of a negative *U*_eff_ — although not physically interpretable as a Coulomb interaction — serves as a pragmatic way to align the DFT DOS with the DMFT DOS (see Figures 1c; S1 in Section S1, Supporting Information). As shown in the middle and right panels of Figure 1c, where a value of *U*_eff_ = −3.1 eV is employed, this approach significantly reduces the spin splitting and shifts the Fe *d*‐states closer to the Fermi level, sharpening their spectral features. As detailed in the supporting information (Sections S1 and S9, Supporting Information), several *U*_eff_ values were tested, and *U*_eff_ = ‐3.1 eV was selected as the best compromise: it reproduces the narrowed Fe *d*‐band DOS obtained from DMFT while maintaining a realistic energy alignment with the molecular orbitals and the experimentally observed hybridization strength.

The effectiveness of DFT+U with negative *U*_eff_ may be attributed to the Fermi‐liquid nature of the substrate. Near the Fermi level, the imaginary part of the dynamical self‐energy is negligible, and the value of *U*_eff_ effectively serves as a fitting parameter for the real part of the self‐energy, which induces a shift of the states. In this sense, the approach resembles a scissor‐operator, commonly employed to align Kohn–Sham states with quasiparticle energies.^[^ ^40^ ^]^ Naturally, the agreement between DFT+U (with negative *U*_eff_) and DMFT deteriorates away from the Fermi level. Incoherent correlation effects, such as satellite features, remain uncaptured, as they require an energy‐dependent self‐energy (including non‐negligible real as well as imaginary parts) rather than a rigid shift of the band structure. Nevertheless, these features emerge well below the Fermi level in our system^[^ ^23^, ^41^ ^]^ and are thus expected to play only a secondary role in chemical bonding.

Far more relevant for chemical bonding, however, are the exchange splitting and the width of the Fe *d*‐bands, which fundamentally shape the predictions of chemisorption models describing the interaction between the Fe–O surface and a model adsorbate state, as illustrated in Figure 1b. According to the *d*‐band model, the discrete adsorbate level first broadens and shifts down in energy due to interaction with the substrate's delocalized *sp*‐electrons. Next, if there is sufficient energy overlap between the renormalized adsorbate states and the modified metal *d*‐bands, and a considerable wave function overlap exists, this interaction leads to the formation of bonding and antibonding states between the metal and molecule.

In the following, we combine STM and STS measurements with POT to demonstrate that the reduced spin splitting of the *d* states, induced by electron correlation at the Fe–O surface, and mimicked with DFT+U (using negative *U*_eff_) enhances both the energetic and spatial overlap with the frontier MOs. This promotes covalent coupling and drives the transition from weak physisorption to strong chemisorption.

### 2.2 Pentacene on Oxygen‐Passivated Iron: Geometric and Electronic Structure

**Figure 2:**
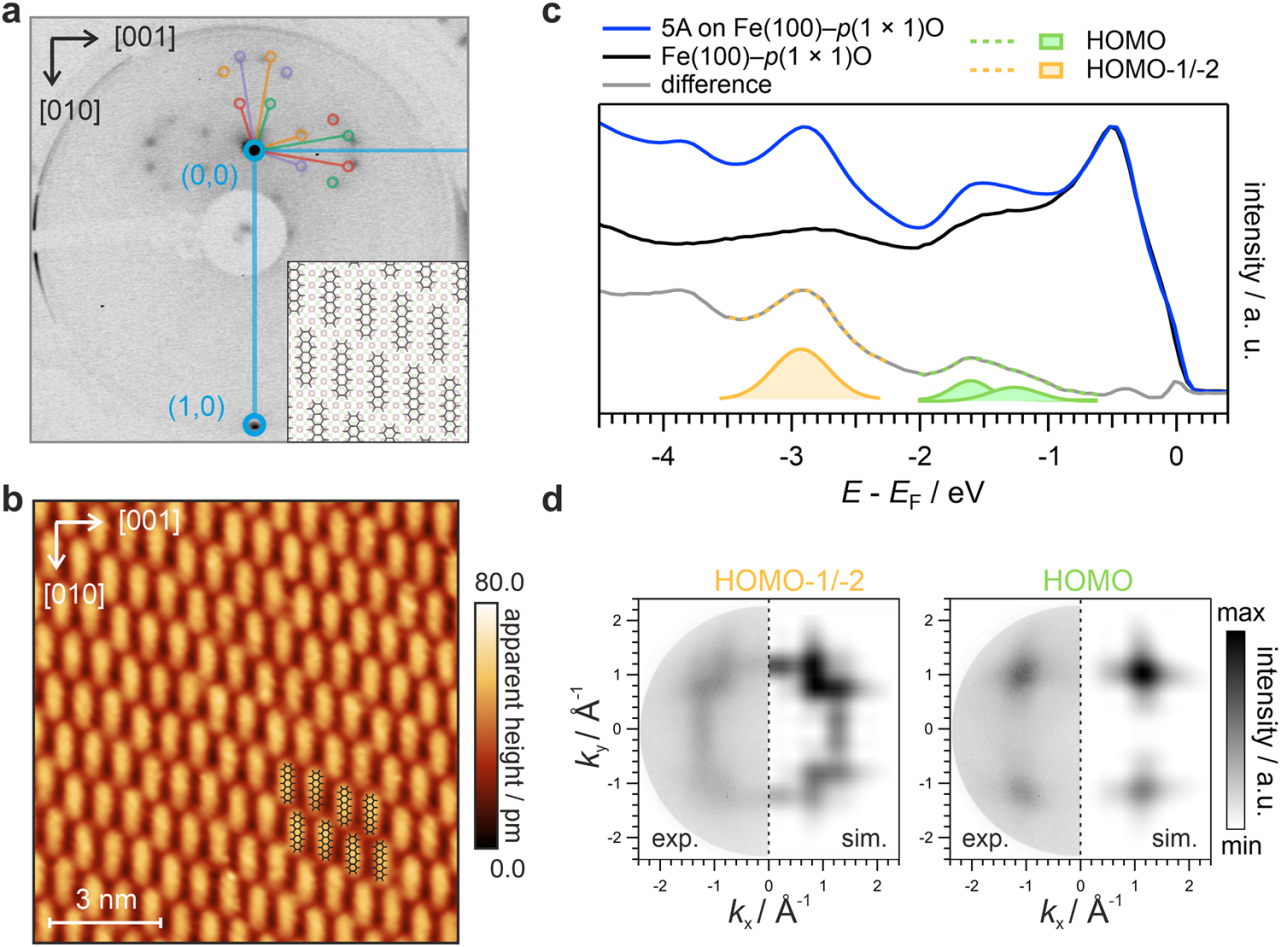
Structural and electronic properties of 5A adsorbed on Fe(100)–p(1 × 1)O. a) LEED image (E_kin_ = 24 eV) of one saturated monolayer of 5A atop Fe–O. The simulated pattern of the 5A superstructure is superimposed. The inset at the bottom right shows the corresponding real space alignment of the molecules within a single rotational domain. b) STM image showing a single 5A rotational domain atop the Fe–O, (V_bias_: 2.0 V, I_tun_: 200 pA). c) Momentum‐integrated photoemission spectra of both the clean Fe–O and the 5A‐covered Fe–O, taken with 40 eV photon energy. The two highlighted peaks at −1.6 and −2.8 eV correspond to the HOMO and HOMO−1/−2 of 5A, respectively. d) Momentum‐resolved photoemission maps of the HOMO−1/−2 (left) and HOMO (right) peaks juxtaposed to the simulated photoemission patterns of the respective orbitals.

We begin by examining the structural properties of the 5A molecules on the passivated Fe–O surface. As the low energy electron diffraction (LEED) image in **Figure** **2**a demonstrates, deposition of a saturated monolayer (1 ML) of 5A results in a long‐range ordered molecular film. In addition to the substrate‐related diffraction pattern (blue), we identify the presence of four symmetry‐equivalent molecular domains whose diffraction spots are clearly visible on the reported LEED image.

We find that one of these domains can be described by a commensurate (3, 1; −1, −6) superstructure. Despite the occurrence of four different molecular unit cells, the STM images reveal only two azimuthal orientations of the 5A molecules, which align with their long axis either parallel to the [010] or [001] substrate axes. Combining the LEED and STM data, we can deduce a structural model, which is depicted at the bottom panel of Figure 2a.

To gain detailed insight into the interfacial electronic structure, we perform photoemission measurements of the 5A monolayer in the valence band region. Ultraviolet photoelectron spectroscopy (UPS) data of the clean Fe–O substrate and the 5A/Fe–O interface are shown in Figure 2c (black and blue curves, respectively). The UPS spectra are obtained by integrating the photoelectron intensities over a wide momentum range and plotting the resulting signal as a function of the energy with respect to *E*_F_. From this comparison, we identify two prominent peaks in the 5A/Fe–O spectrum at approximately −1.6 and −2.8 eV below the Fermi level. These energies provide initial estimates for the molecular states involved, but an unambiguous assignment requires access to momentum‐resolved information.

By utilizing POT,^[^ ^42^, ^43^, ^44^ ^]^ we analyze the full momentum distribution of photoelectrons and assign these features to specific molecular orbitals. In Figure 2d, we compare the experimental momentum maps at selected energies to theoretical reference maps generated from gas‐phase DFT orbitals of isolated pentacene. The simulation procedure of the momentum maps, including domain averaging and polarization effects, is outlined in Figure S2 (see Section S2, Supporting Information). The resulting excellent agreement between experiment and simulation allows us to attribute the first peak at −1.6 eV to the highest occupied molecular orbital (HOMO) and the second peak at −2.8 eV to a combined contribution of the HOMO−1 and HOMO−2, even though substrate‐related features exhibit a similar momentum pattern as the HOMO (see Figure S3 in Section S3, Supporting Information). Notably, the agreement with gas‐phase calculations in POT is a commonly observed behavior that even holds in the strong chemisorption regime, as the nodal structure of the orbital pattern is a robust property that is generally preserved upon adsorption.^[^ ^43^ ^]^

To further analyze these valence band features quantitatively and to disentangle substrate‐related features from the contribution of the 5A molecules, we follow established methods^[^ ^24^, ^44^ ^]^ and subtract the background coming from the Fe–O substrate, which results in the grey curve in Figure 2c. The spectra are normalized to the intensity around the Fermi energy, *i.e*. the region [0.0, −0.5 eV], where no discernable influence of the molecules is observed in the momentum‐resolved data (see Figure S4 in Section S3, Supporting Information for details). To extract the quantitative information, the corresponding peak regions were fitted using Gaussian functions with a linear background. The latter accounts for the baseline rise from inelastic scattering and secondary electron contributions, which vary upon molecular adsorption and are therefore not fully removed by subtracting the clean‐substrate spectrum. The combined HOMO−1/HOMO−2 feature is accurately described by a single Gaussian peak at (−2.88 ± 0.01) eV with a full width at half maximum (FWHM) of (0.52 ± 0.01) eV. However, to properly describe the HOMO‐related emission, two Gaussian contributions are required, which are centered at (−1.26 ± 0.05) eV and (−1.61 ± 0.02) eV, with FWHMs of (0.46 ± 0.04) eV and (0.35 ± 0.02) eV, respectively (see Section S4, Supporting Information for details on the fitting procedure). Notably, our momentum‐resolved data allow us to rule out any contributions from the lowest unoccupied molecular orbital (LUMO) in the energy window below *E*_F_, indicating that no electron transfer occurs from the substrate to the organic adlayer. This behavior contrasts with recent observations of electron transfer for cobalt tetraphenylporphyrin molecules adsorbed on the Fe–O surface.^[^ ^45^ ^]^

**Figure 3:**
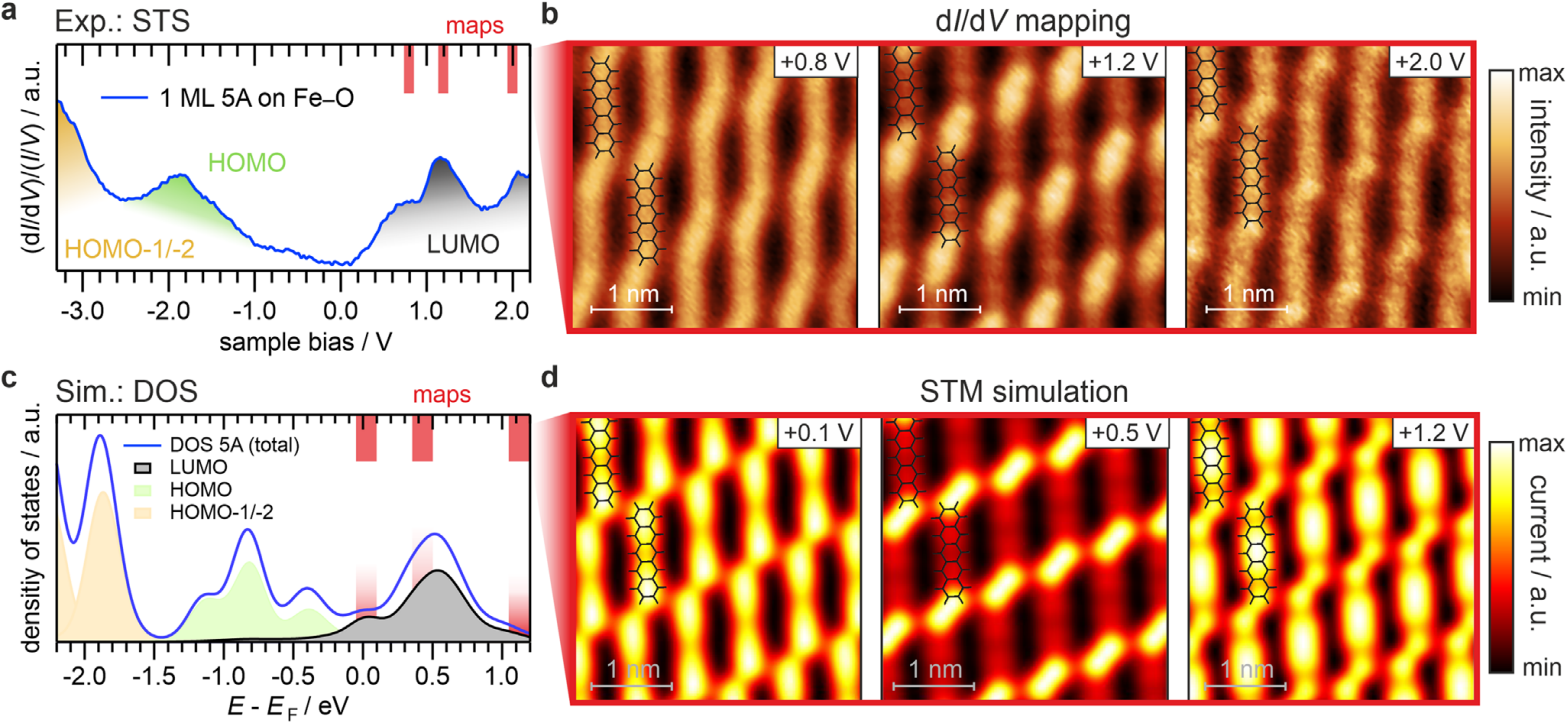
STS data of the occupied and unoccupied states of 5A atop the Fe–O surface. a) Normalized dI/dV spectrum measured on top of a single 5A molecule. b) dI/dV maps obtained for a tunneling current of 300 pA at different positive voltages close to the peaks observed in the unoccupied region (red bars), each probing a 3.5 x 3.5 nm*^2^* area. c) MO‐resolved PDOS for 5A adsorbed on Fe–O derived from DFT+U calculations using a Hubbard‐parameter of U_eff_ = −3.1 eV. d) Simulated STM images in correspondence to the experimentally obtained images from Figure 3b. Images are obtained by integrating over 150 mV near the highlighted regions of the identified peaks (red bars in Figure 3c). The maximum values of these integration ranges are indicated at the top of each panel. STM simulations were performed at a constant height of 4 Å.

To complement our understanding of the frontier orbitals’ electronic structure of the adsorbed 5A molecules, we extend our study to STS experiments, which allow us to additionally probe unoccupied states. In the occupied region of the STS spectrum (**Figure** **3**a, negative *V*_bias_), the two prominent peaks at roughly −1.8 and −3.2 V align well with the corresponding ones from our photoemission data. The previous assignment to the HOMO and HOMO−1/HOMO−2 is further confirmed by STM measurements (Figure S9c, Supporting Information). Moreover, the HOMO resonance in STS exhibits a similar broadened shape, like the one obtained through UPS.

Interestingly, the unoccupied states (Figure 3a, positive *V*_bias_) reveal a more textured and complex structure. Instead of a single, potentially broadened LUMO resonance, two distinct peaks appear at 0.8 and 1.2 V, accompanied by a less pronounced peak at 2.0 V. This is in stark contrast to the commonly reported scenario of weakly interacting molecules on passivated surfaces, where charge transfer and hybridization are inhibited.^[^ ^46^ ^]^ Here, the LUMO usually gives rise to a single resonance, situated well above the Fermi energy.^[^ ^47^, ^48^, ^49^ ^]^ Both the broadening of the HOMO and the appearance of several LUMO‐like features suggest the involvement of more complex interactions that warrant further investigation.

First, we exclude intermolecular interactions as the origin for the sub‐structure in the LUMO, by performing DFT calculations for a free‐standing 5A layer, which do not indicate any significant intermolecular dispersion effects (see Figure S11, Supporting Information). Second, a control experiment with only a half‐monolayer (0.5 ML) 5A film, where the molecules arrange in a different unit cell, exhibits the same spectroscopic fingerprints (see Section S5, Supporting Information). Last, the higher energy structure in the unoccupied STS is also most likely not due to the LUMO+1, which would be expected at considerably larger energies. This can in fact be evidenced by recording 2D conductivity maps — referred to as d*I*/d*V* maps — near the relevant bias voltages, indicated by red bars in Figure 3a. The d*I*/d*V* mapping approximates the local electron density distribution at specific bias voltages, which enables the identification of MO fingerprints at selected binding energies. The resulting maps of the three mentioned features in the unoccupied region are presented in Figure 3b, each displaying stripe‐like patterns, however, with distinct intensity variations. While the first map at 0.8 V reveals molecular states characterized by stripes with a slight dip in the center, the second peak at 1.2 V displays a very pronounced intensity profile, featuring a significantly attenuated central region and prominent protrusions at the ends of the molecules. In contrast, the last peak at 2.0 V shows an almost homogeneous intensity profile along the entirety of the individual 5A molecules for both the 1 ML and 0.5 ML film (see Figure S12, Supporting Information for a comparison of line profiles). To verify that the presented d*I*/d*V* maps indeed originate from the LUMO, we performed high‐resolution STM measurements on both the 1 ML and the 0.5 ML film of 5A adsorbed on Fe–O (see Figure S9, Supporting Information). In the corresponding images, the molecules appear as 7‐lobe structures (up to a *V*_bias_ of 2.0 V), each resembling the spatial distribution of the LUMO charge density, without any spectroscopic fingerprint of the LUMO+1 being present (see Figure S9b, Supporting Information). Strikingly, we can account for these experimental observations by simulating the electronic structure of the full 5A/Fe–O interface by means of our DFT+U calculations with the tailored *U*_eff_ = −3.1 eV. The resulting DOS projected on the 5A molecules (PDOS) is displayed in Figure 3c as blue curve, alongside the molecular‐orbital‐projected DOS (MOPDOS) of the frontier orbitals, which dominate the presented region. Overall, the shape of the simulated PDOS shows a strong resemblance to the experimental spectrum. Although the energy separation between the HOMO and the Fermi level, as well as between the LUMO and the Fermi level, is underestimated (likely due to limitations of our approach combined with the well‐known underestimation of molecular bandgaps in standard DFT^[^ ^50^ ^]^) a one‐to‐one assignment of the peaks in the calculated PDOS to the experimental spectrum can be achieved. To substantiate this, we simulated the constant‐height STM patterns at the three LUMO peak positions highlighted by red bars in Figure 3c. The resulting simulations closely replicate the essential intensity variations in the experimental d*I*/d*V* maps, thereby reinforcing their assignment as LUMO‐related features. A similar conclusion can be drawn by comparing the measured d*I*/d*V* maps with the calculated electron densities at the corresponding energies, as shown in Section S8 (Figure S13, Supporting Information).

Having already excluded intermolecular interactions, this overall agreement between experiment and theory gives us confidence that the peculiar appearances of the peaks of the frontier orbitals are indeed an interface‐related effect. In the following, we will focus on the interpretation of the theoretical results, thereby shedding light on the critical role of the substrate–molecule interaction, and the key mechanisms that determine the chemical bond formation.

### 2.3 The Importance of Capturing the Substrate *d*‐Bands

The overall good agreement between the experimental data and the theoretical results underscores the importance of accurately describing the surface *d*‐band structure to reproduce the molecular chemisorption in our system. This becomes evident when comparing the outcomes of standard DFT (*U*_eff_ = 0 eV) with those of DFT+U (*U*_eff_ = −3.1 eV).

**Figure 4:**
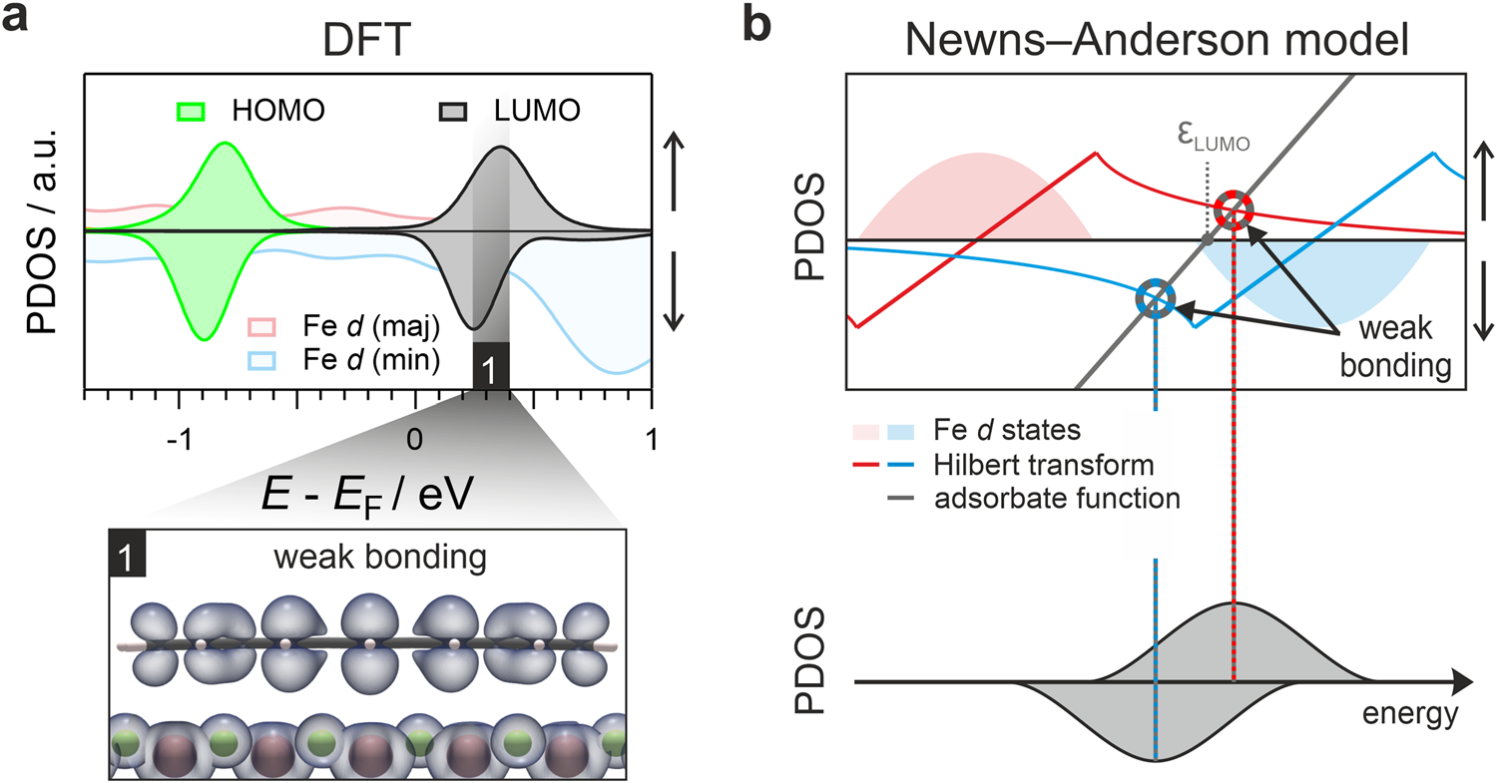
DFT‐based analysis of 5A adsorption on Fe–O and the spin‐resolved Newns–Anderson model. a) PDOS showing the HOMO and LUMO contributions alongside the Fe d‐bands resulting from DFT calculations of 5A adsorbed on top of Fe–O. The bottom panel shows a plot of the electron density stemming from the energy interval [0.2, 0.4] eV, which marks the center of the LUMO peak indicated by the black bar. b) Sketch of the spin‐resolved Newns–Anderson model assuming a Fe d‐band structure similar to the one obtained by DFT. Solid red and blue lines indicate the Hilbert transforms of the Fe majority and minority PDOS, respectively. The grey dotted line indicates the energy position E_LUMO_ of the renormalized LUMO state, from where the adsorbate function, *y(E) = E−E_LUMO_* emerges (solid grey line). The intersections between the adsorbate function and the Hilbert transforms (highlighted by circles) mark the energy positions of the hybridized adsorbate–metal states. In the presented case, the model predicts a single resonance in each of the two spin channels (see bottom panel).

Standard DFT yields a simple DOS, as illustrated in Figure 1a. The Fe *d*‐bands exhibit a pronounced Stoner‐type spin splitting of ≈ 3.0 eV (see also Figure 1d), resulting in a significant energetic mismatch between the *d*‐states and the frontier MOs of 5A. Consequently, little chemical bonding is observed, as evidenced by the spin‐resolved MOPDOS for the 5A/Fe–O interface shown in **Figure** **4**a, which displays only moderate broadening of the HOMO and LUMO features in both spin channels. Correspondingly, the electron density integrated over the LUMO energy window (marked by the black bar labeled (1) in Figure 4a) closely resembles that of the gas‐phase LUMO (see Figure S9b, Supporting Information).

**Figure 5:**
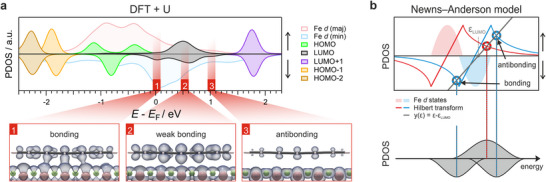
DFT+U‐based electronic structure of the 5A/Fe–O interface and the impact of d‐band modifications. a) Resulting PDOS for 5A adsorbed on Fe–O, obtained from optimized DFT+U calculations using U_eff_ = ‐3.1 eV. The adsorption geometry is the same as in Figure 4a. For the MOPDOS of the LUMO we identify three main peak regions, which are indicated by red bars. In the bottom part the corresponding electron densities (spin‐integrated), which stem from the highlighted peak regions, are plotted. b) Scheme of the spin‐resolved Newns–Anderson model for a substrate DOS like the one from Figure 5a, including electron correlation. In the top graph, the adsorbate function (solid grey line) intersects the majority Hilbert transform (solid red line) only once, while three intersections are found between the adsorbate function and the Hilbert transform of the minority states (blue line). According to the model, this results in a single broadened resonance in the spin‐up PDOS, and the formation of bonding and antibonding states in the spin down.

In contrast, the DFT+U approach presents a markedly different scenario (for *U*_eff_ = −3.1 eV; a systematic analysis of dependence of the results on *U*_eff_​ is provided in Section S9, Supporting Information). As shown in the spin‐resolved MOPDOS in **Figure** **5**a, two pronounced overlaps emerge: the HOMO aligns with the Fe majority spin states, while the LUMO overlaps with the Fe minority spin states. This enhanced energetic and spatial overlap — enabled by the reduced exchange splitting of the Fe–O surface, induced by electron correlation and mimicked through the negative *U*_eff_​ potential — promotes strong covalent coupling and drives the system from weak physisorption into a chemisorbed regime.

These findings can also be interpreted within the framework of the Newns–Anderson model, and its extension, the *d*‐band model. In these frameworks, the spin‐dependent interaction between a renormalized LUMO and the *d*‐bands of a metallic substrate can be understood by analyzing the intersections between the Hilbert transform of the substrate's spin‐resolved *d*‐PDOS and the energy‐dependent adsorbate function, defined as, *y*(*E*) = $y\left(E\right)=\frac{1}{V^{2}}\left(E-E_{\mathrm{LUMO}}\right)$(*E−E*_LUMO_). Here, *E*_LUMO_ is the energy of the renormalized LUMO, *i.e*., its position after interaction with the delocalized *sp*‐states of the substrate. *V* ^2^ denotes the adsorbate–metal‐*d* coupling strength,^[^ ^7^, ^12^ ^]^ which depends on the spatial overlap between the adsorbate state and the metal *d*‐states.^[^ ^14^ ^]^ The slope of the adsorbate function is inversely proportional to the coupling strength, meaning steeper slopes correspond to weaker coupling. The number and position of the intersections between the adsorbate function and the Hilbert transform provide two sets of information: a single intersection indicates mere broadening of the adsorbate resonance, while three intersections signal a splitting into bonding and antibonding states. Of these three intersections, only the outer ones correspond to the actual bonding and antibonding states, while the middle one represents a weak, non‐localized resonance with negligible spectral weight.^[^ ^7^, ^14^ ^]^

Applying this model to our system and assuming a coupling strength of *V* ^2^ = 1 eV, we find that when using the *d* band position obtained by standard DFT (*U*_eff_ = 0), the LUMO exhibits only a single intersection with the Hilbert transform in both spin channels. This indicates mere energetic broadening, without the formation of distinct bonding and antibonding features — consistent with the MOPDOS results shown in Figure 4a. In contrast, when employing DFT+U, the *d*‐bands are brought into much closer energetic proximity to the LUMO. Then, using the previously introduced LUMO adsorbate function and considering its coupling to the now narrower *d*‐bands, we observe a markedly different behavior. In the spin‐down channel, where the renormalized *E*_LUMO_ lies within the energy range of the substrate *d*‐bands, the condition for multiple intersections is fulfilled, resulting in the emergence of separated bonding and antibonding states (see Figure 5b). Conversely, in the spin‐up channel, *E*_LUMO_​ remains outside the *d*‐band manifold, resulting in only a single intersection. This corresponds to a broadened, non‐hybridized molecular resonance rather than a true hybrid state. Thus, the adsorption mechanism predicted by the Newns–Anderson model is consistent with the MOPDOS results shown in Figure 5a, thereby highlighting the essential role of correlation‐induced *d*‐band renormalization in facilitating strong chemisorption.

A closer inspection of the integrated charge density in the LUMO region from the DFT+U calculation further supports the predictions derived from the *d*‐band model. The spin‐integrated density plots shown in the bottom of Figure 5a indicate that each of the peaks corresponds to a distinct bonding character. The peak closest to the Fermi level (labeled (1)) displays a strong bonding interaction between the three central LUMO lobes and the Fe *d*‐states, consistent with the predicted bonding state of the spin‐down channel in the Newns–Anderson *d*‐model. Conversely, the peak farthest from the Fermi level (labeled (3)) exhibits a clear antibonding character, again in line with the expected model behavior for the spin‐down channel. In contrast, the central peak (labeled (2)), which contributes most strongly to the spectral weight, corresponds to a weakly bonding or non‐hybridized state, closely resembling the electronic character observed in the uncorrected DFT case (*U*_eff_ = 0).

To explore the broader implications of our findings, we replaced 5A with atomic nitrogen (N) — a catalytically relevant adsorbate — and placed it atop the Fe–O surface (see Section S10, Supporting Information). Our results demonstrate that including the correlation‐induced changes in the *d*‐band structure within the DFT+U approach, leads to a significant increase of the N chemisorption energy, with respect to the single‐particle picture. Noteworthy, this observed trend is consistent with the recent reports of enhanced catalytic activity at 3*d* FM surfaces upon quenching their magnetic properties.^[^ ^16^, ^17^ ^]^ These observations reinforce our interpretation based on the *d*‐band model and the importance of correlation‐driven *d*‐band modifications for the understanding of chemical bonding at interfaces that involve FM TM surfaces.

It is important to note, however, that while the spin‐resolved Newns–Anderson model qualitatively accounts for the emergence of the three LUMO resonances measured in the spin‐integrated STM/STS data, it does not fully capture the orbital splitting in the spin‐down channel predicted by the DFT+U calculations — specifically, the appearance of three, rather than two, LUMO‐related resonances (see Figure 5a). This is somewhat unexpected, especially when contrasted with the HOMO in the spin‐up channel (also Figure 5a), which, despite undergoing equally strong chemical interactions, exhibits only two distinct resonances in line with the simplified model's predictions. In the next section, we will examine a possible origin of this discrepancy.

### 2.4 Mechanisms of Surface Chemical Bonding

Having addressed the energetic alignment between the molecule's LUMO and the substrate *d*‐bands, we now turn to the second crucial factor governing chemical bond formation: the spatial overlap between the adsorbate and substrate states, represented by the coupling strength, *V* ^2^. For larger organic systems such as 5A, the adsorption site plays a pivotal role in modulating this spatial overlap. Specifically, on Fe–O, shifting 5A molecules by half a substrate unit cell vector relative to the optimized adsorption geometry results in the absence of split molecular resonances or other signs of enhanced chemical interactions (see Section S11, Supporting Information). This absence is attributed to a reduction in the spatial overlap between molecular and metal states, which weakens the coupling between the Fe *d*‐states and the frontier MOs.

**Figure 6:**
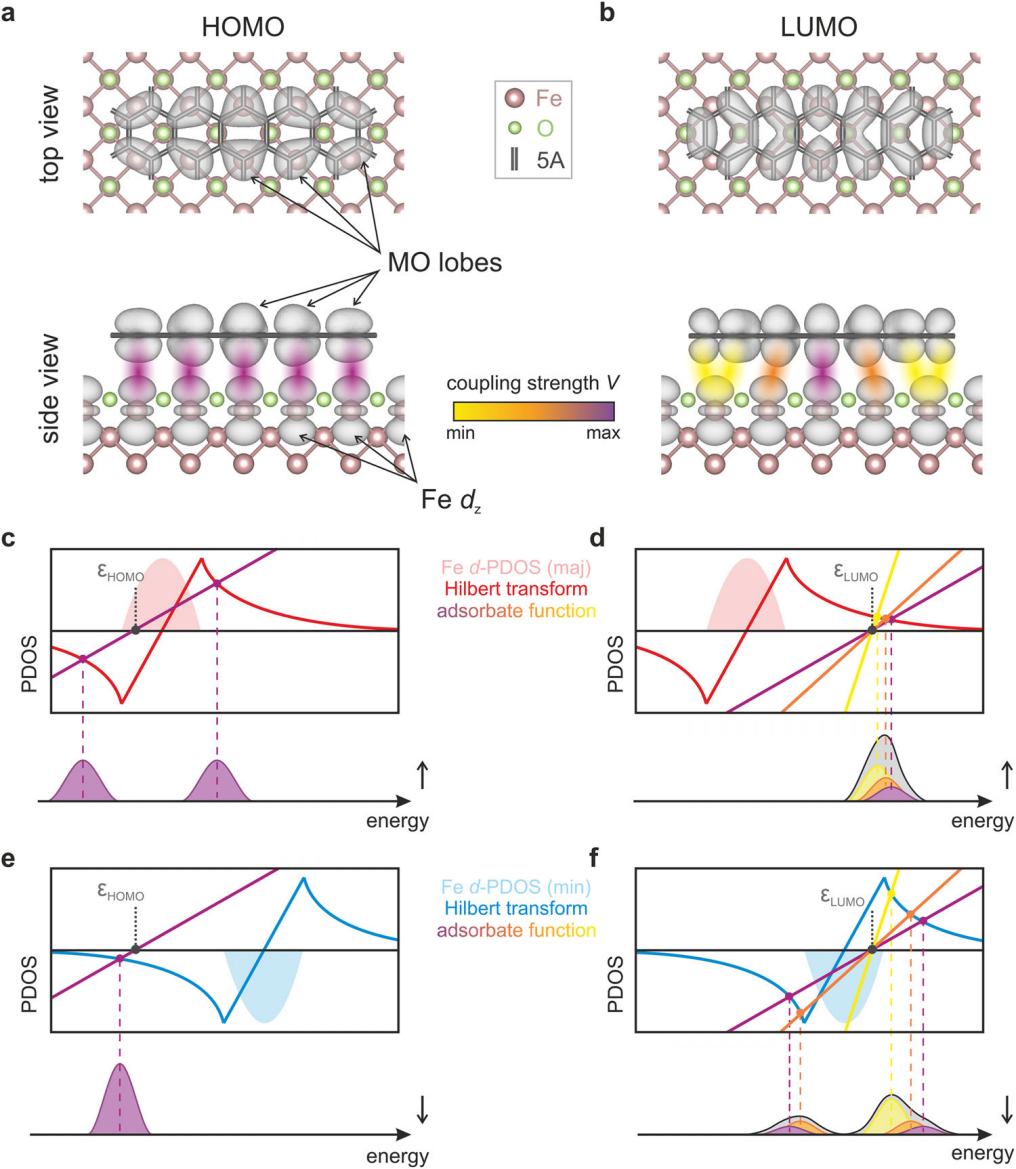
Schematic of the bond formation between the 5A frontier orbitals and the Fe d‐states. Plots of the gas phase a) HOMO and b) LUMO of 5A placed atop the Fe–O surface for the optimized adsorption geometry. In the bottom panels, the assumed spatial overlap between the MO lobes and idealized Fe d orbitals is highlighted by the indicated color code. c,d) Newns–Anderson model for the interaction of the 5A c) HOMO and d) LUMO with the Fe majority d‐states. In accordance with the model, the interaction of the HOMO is approximated by a single adsorbate function with slope s = 1/V^2^ = 0.5, while the non‐uniform degree of overlap between the Fe and LUMO orbital lobes, results in a set of adsorbate functions with varying slopes of s = 0.5, s = 0.8, and s = 2.4. In the bottom panels, estimations of the resulting MOPDOSs are presented. e,f) Presentation of the Newns–Anderson model, describing the interaction between the minority Fe d‐states and the e) HOMO or f) LUMO.

To illustrate this point, we present the electron densities of the gas phase HOMO and LUMO of 5A atop the Fe–O surface in the optimized adsorption configuration (**Figure** **6**a,b, respectively). The HOMO lobes are almost perfectly commensurate with the Fe *d*‐states of the substrate, due to a spatial coincidence of its lobe structure and the Fe surface lattice (Figure 6a). In contrast, the LUMO, which exhibits a shorter spatial periodicity, shows significant overlap primarily in its central lobes. Toward the molecular edges, the overlap with Fe *d*‐states progressively weakens (Figure 6b). This spatially non‐uniform coupling between the LUMO and the Fe surface presents a challenge to the traditional *d*‐band model, which assumes a uniform interaction across the entire molecule.

To remain within the conceptual framework of the *d*‐band model while accounting for spatial inhomogeneities in molecule–substrate interactions, we propose an empirical extension that incorporates local coupling strengths. Specifically, we assume that different regions of the molecular orbitals interact locally with the underlying Fe atoms, allowing the *d*‐band model to remain applicable when adapted to spatially resolved interactions. To this end, we spatially partition the LUMO into a central, middle, and outer region indicated by purple, orange, and yellow in Figure 6b, respectively. By approximating the local coupling between these confined LUMO regions and the Fe *d*‐band as an interaction between C *p*_z_ and Fe d_z_^2^ orbitals, weighted by a geometry factor (details in Section S12, Supporting Information), we obtain different effective coupling strengths *V* ^2^ of 2.0, 1.6, and 0.4 eV for the central, middle, and outer regions, respectively. Notably, the extracted coupling strengths fall within the typical range reported in the literature for adsorbates on TM surfaces.^[^ ^13^, ^14^, ^51^ ^]^ For the HOMO, only one effective coupling strength of *V* ^2^ = 2.0 eV results from the same procedure, reflecting its consistent overlap with the Fe lattice.

Finally, we convert the calculated coupling strengths into slope values for the different lobe functions. The resulting spin‐resolved analyses are shown in Figure 6c–f for the HOMO and LUMO, respectively. Following the standard procedure described in the previous section, the resonances are determined from the intersections of the respective lobe functions with the Hilbert transforms. For the HOMO, this yields two peaks in the spin‐up channel and one in the spin‐down channel, consistent with both the DFT+U results and STM measurements (see Figure S18 in Section S13, Supporting Information). In contrast, the situation is more complex for the LUMO case owing to the prediction of three different local coupling strengths, hence slopes. In the spin‐up channel, the energy positions of these intersection points are so close to one another that the resulting resonances effectively merge into a single broadened peak (Figure 6d). In contrast, in the spin‐down channel, the well‐separated intersection points give rise to three distinct peaks (see Figure 6f), which strongly resemble the MOPDOS obtained from DFT+U calculations. Thus, according to this interpretation, we conclude that the observed orbital splitting is not only sensitive to the *d*‐band spin‐splitting and width, but also to the registry of the MO's lobe structure on the underlying Fe lattice, which can produce a significantly more complex DOS than predicted by simplified uniform‐coupling models.

## 3 Conclusion and Outlook

In conclusion, our combined theoretical and experimental investigation of 5A molecules adsorbed on oxygen‐passivated Fe(100) demonstrates that simplified chemisorption frameworks can be meaningfully extended to complex metal–adsorbate systems, even when electron correlation plays a significant role. Standard DFT fails to reproduce the observed bonding characteristics at the interface, whereas a modified approach based on DFT+U — incorporating correlation‐induced *d*‐band renormalization via a tailored negative *U*_eff_ — successfully captures the key spectroscopic signatures seen in experiment.

Our results highlight that the reduction of spin splitting and narrowing of the *d*‐band, both driven by many‐body interactions, are decisive factors in determining the strength and nature of molecule–metal bonding at 3*d*‐ferromagnetic surfaces. A comparative analysis using atomic nitrogen further confirms that these changes can substantially enhance chemisorption energies, underscoring their potential impact on surface reactivity and catalytic performance on correlated metal surfaces.

To bridge the gap between simplified models and complex organic adsorbates, we extend the *d*‐band model by introducing orbital lobe functions that account for the spatially modulated overlap between molecular orbitals and metal *d*‐states. This refined framework captures electronic fingerprints and bonding characteristics that elude conventional approaches, offering an intuitive and physically grounded route to describing molecule–surface interactions and providing transferable design rules for extended organic adsorbates on transition‐metal substrates.

While further experimental and theoretical validation will be important to assess the broader applicability of this framework, our findings establish correlation‐driven *d*‐band renormalization as a key concept for understanding and ultimately controlling adsorption phenomena at correlated metal interfaces.

By explicitly linking correlation‐induced modifications of the *d*‐band structure to chemisorption energies and spin‐dependent hybridization, the present approach suggests concrete strategies for tuning adsorption at ferromagnetic surfaces, with direct implications for the design of more active 3*d*‐metal catalysts and for engineering charge and spin injection in organic electronic and spintronic devices.

## 4 Experimental Section

### Sample Preparation

The Fe(100) substrate was prepared starting from a commercial MgO(100) crystal (MaTecK), which was used as support substrate. The MgO sample was transferred into an ultra‐high‐vacuum (UHV) system with a base pressure below 7 × 10^−11^ mbar and cleaned via repeated cycles of Ar⁺ sputtering at 2 keV for 30 min, followed by annealing at 870K for 1 h. Subsequently, a 300 nm Fe film was epitaxially grown in situ by e‐beam deposition on the cleaned MgO surface, maintained at room temperature. The freshly grown Fe(100) film was flash‐annealed at 870K to improve the surface quality. For each preparation, the Fe surface was cleaned by gentle Ar⁺ sputtering at 0.5 keV for 10 mi, followed by annealing at 870K for 5 min. The Fe(100)–*p*(1 × 1)O surface was prepared by exposing the clean Fe(100) surface to an O_2_ atmosphere at 1.3 × 10^−7^ mbar for 5 min (30 L) while the sample temperature was kept at 820K. Excess oxygen was removed by annealing at 900K for 5 min. After each preparation step, the sample cleanliness and surface quality were monitored using Low‐Energy Electron Diffraction (LEED) and Auger Electron Spectroscopy (AES).

To create the 5A/Fe–O interfaces, pentacene (5A) molecules were evaporated from a water‐cooled, Knudsen‐cell‐type evaporator (Kentax GmbH). The deposition rate was pre‐calibrated with a quartz crystal microbalance and confirmed by monitoring the emerging LEED pattern during stepwise deposition. The growth rate at 458K was estimated as 0.125 ML min^−1^, referenced to a full saturated monolayer (1 ML). The 0.5 and 1 ML 5A/Fe–O interfaces were achieved by depositing 5A on freshly prepared Fe–O substrates for 4 and 8 min, respectively, with the sample held at room temperature.

The sample preparation was performed independently at the TU Dortmund University, Germany, and the NanoESCA beamline at the Elettra synchrotron, Italy. Both preparation chambers are equipped with LEED and AES instruments and are coupled to a Photoemission Electron Microscope (PEEM). The Kentax evaporator was consistently used across setups, ensuring similar 5A deposition rates. Thermal removal of adsorbed pentacene layers was achieved by flash‐annealing the sample at 720–770K.

### Momentum‐Resolved Photoemission Measurements

Momentum‐resolved photoemission data of the 1 ML 5A film (Figure 2c,d) were collected at the NanoESCA beamline at Elettra, utilizing a NanoESCA PEEM (Focus GmbH) in momentum mode with *p*‐polarized light at 40 eV photon energy. During the measurements, the sample temperature was maintained at 80K, and the sample was continuously rastered to minimize radiation‐induced damage. The analyzer's energy resolution was estimated to be below 100 meV.

Additional momentum‐resolved experiments were conducted in Dortmund for both 1 and 0.5 ML films to verify sample quality before transferring to the STM machines. Measurements were performed at room temperature using a Kreios PEEM (Specs GmbH) coupled to a laser‐based light source providing *p*‐polarized fs‐XUV pulses at 29.7 eV.^[^ ^52^ ^]^ The energy resolution was below 300 meV.

Both momentum microscopes allowed the recording of 2D momentum maps at constant kinetic energies, with a field of view of up to *k*_x_, *k*_y_ ∈ [−2.5, 2.5] Å^−1^. Data analysis was conducted using the Igor Pro 8 software by Wavemetrics.

### Sample Transfer to STM Chambers

The 5A interfaces for STM studies were prepared and characterized at TU Dortmund, with initial analyses including LEED and photoemission. Samples were then transferred to the STM systems using a Ferrovac UHV suitcase, maintaining a base pressure below 5 × 10^−11^ mbar during transfer. The suitcase was connected to the STM chambers via fast‐entry load locks, which were baked and allowed to cool down before sample transfer. During transfer into the load lock, samples were briefly exposed to pressures of ≈ 5 × 10^−8^ mbar. The entire process from preparation to transfer took 2–5 days.

### STM and STS

STM/STS experiments on the 0.5 ML 5A film were performed at the University of Duisburg–Essen, Germany, using a home‐built low‐temperature STM at 80K and a base pressure of 5 × 10^−10^ mbar. Topographic STM images were acquired at constant tunneling current, with the bias voltage (*V*_bias_) applied to the sample. Differential conductance (d*I*/d*V*) images were recorded at constant height using the lock‐in technique.

STM/STS measurements on the 1 ML 5A film were conducted using an Omicron low‐temperature STM at CNR – Istituto Officina dei Materiali (IOM) in Trieste, Italy, under UHV conditions (base pressure < 7 × 10^−11^ mbar) at 77K. Topographic STM images were captured in constant‐current mode, and d*I*/d*V* images were recorded at constant height using the lock‐in method.

All STM and d*I*/d*V* images were analyzed using the Gwyddion software package. The STS spectra were processed with Igor Pro 8 (Wavemetrics).

### DFT and DFT+U Calculations

The periodic calculations have been performed with the Vienna Ab Initio Simulation Package (VASP) version 5.4.4.^[^ ^53^, ^54^ ^]^ The projector‐augmented wave (PAW) method^[^ ^55^ ^]^ assuming an energy cutoff of 400 eV was used. Exchange‐correlation effects were approximated by the functional of Perdew–Burke–Ernzerhof (PBE)^[^ ^56^ ^]^ Van der Waals contributions were treated with Grimme's D3 dispersion correction.^[^ ^57^ ^]^ Additionally as described in the manuscript, an effective Hubbard‐U correction^[^ ^58^ ^]^ was included to the *d*‐states of the upmost Fe‐layer (*U*_eff_ = −3.1 eV) to capture the electronic changes in the Fe‐substrate upon passivation. The results with the chosen *U*‐Parameter were validated against DMFT calculations,^[^ ^23^ ^]^ as shown in the Supporting Information. The interfaces were simulated within the repeated slab approach using six substrate layers and a 30 Å vacuum layer between the slabs. The unit cell of the surface was taken from a previous DMFT investigation of the structure^[^ ^23^ ^]^ and held fix, while the ionic positions of the molecules were optimized until forces were below 0.01 eV Å^−1^. To avoid spurious electrical fields, a dipole layer was inserted in the vacuum region.^[^ ^59^ ^]^ For the geometry optimization, the Brillouin zone was sampled on a Gamma‐centered grid of 3 × 5 × 1 k‐points, which was, subsequently, refined to a k‐point grid of 5 × 9 × 1 to evaluate the electronic properties. The STM images are simulated at a constant height of 4 Å based on the Tersoff–Hamann approximation^[^ ^60^ ^]^ assuming a voltage of 0.5 V and a tip radius of 1.5 Å.

## Conflict of Interest

The authors declare no conflict of interest.

## Supporting information



Supporting Information

## Data Availability

The data that support the findings of this study are openly available in [Zenodo Open Research Repository] at [https://doi.org/10.5281/zenodo.17514429], reference number [17514429].
