# Supplementary material for: Correlation‐Driven d‐Band Modifications Promote Chemical Bonding at 3d‐Ferromagnetic Surfaces
Source: Small. 2025 Dec 3;22(13):e08952. doi: 10.1002/smll.202508952 (PMC12954378; doi:10.1002/smll.202508952)
Supplement: Supplementary file 1 — Supporting Information [file SMLL-22-e08952-s001.pdf]

Supplementary Information: Correlation-Driven *d*-Band Modifications Promote Chemical Bonding at 3*d*-Ferromagnetic Surfaces

David Maximilian Janas[1,*,+], Andreas Windischbacher[2,+], Alessandro Sala[3], Vitaliy Feyer[4], Iulia Cojocariu[4], Manuel Gruber[5], Mehdi Bouatou[5], Andrea Droghetti[6], Peter Puschnig[2], Giovanni Zamborlini[1,2,#] and Mirko Cinchetti[1].

[1]*Department of Physics, TU Dortmund University, Dortmund, 44227, Germany.*

[2]*Institute of Physics, University of Graz, NAWI Graz, Graz, 8010, Austria.*

[3]*CNR – Istituto Officina dei Materiali (IOM), S.S. 14 km 163.5, Area Science Park, Basovizza, Trieste, 34149 Italy.*

[4]*Peter Grünberg Institute (PGI-6), Forschungszentrum Jülich GmbH, Jülich, 52425, Germany.*

[5]*Faculty of Physics and CENIDE, University of Duisburg–Essen, Duisburg, 47057, Germany.*

[6] *Department of Molecular Sciences and Nanosystems, Ca' Foscari University of Venice, via Torino 155, Mestre-Venice, 30170, Italy.*

Corresponding authors: * *david.janas@tu-dortmund.de*; # *giovanni.zamborlini@uni-graz.at*

Note: + *these authors contributed equally*

S1: Comparison of the *d*-PDOS at the Fe-O interface for different Hubbard parameter settings

In this section, we closely examine how different theoretical approaches, ranging from Density Functional Theory (DFT) to DFT+U and Dynamical Mean-Field Theory (DMFT), impact the electronic structure of the Fe(100)-*p*(1 × 1)O surface.

Although DFT+U does not capture the full extent of many-body physics, it does allow us to model correlation-induced modifications in the *d*-band structure. To illustrate this, we present a series of Density of States (DOS) calculations simulated with varying U_{eff} Hubbard parameters (see curves i-v in Fig. S1). This comparison reveals that to approximate the DOS obtained by DMFT (vi), negative values for U_{eff} are necessary in the DFT+U simulations. Negative values reduce the *d*-band exchange splitting, which is consistent with the well-known tendency of DFT+U to overestimate the exchange splitting in ferromagnetic transition metal surfaces when positive corrections are applied.

However, a direct comparison between DFT+U and DMFT also highlights the limitations of the DFT+U approach. As it does not incorporate actual many-body physics, DFT+U fails to reproduce spectral features that are purely due to electron correlation. This limitation is particularly evident in the oxygen bands, located in the energy range from -5 eV to -9 eV, which are significantly affected by interactions with so-called satellites—energetically broad features without a clear momentum pattern that arise from many-body interactions. In DMFT, this energy region is dominated by spin-dependent lifetime effects, resulting in broad and smeared-out O 2*p* bands.[1] These spin-dependent effects, intrinsic to many-body interactions, are absent in DFT+U calculations, which consistently produce comparatively narrow O 2*p* bands.

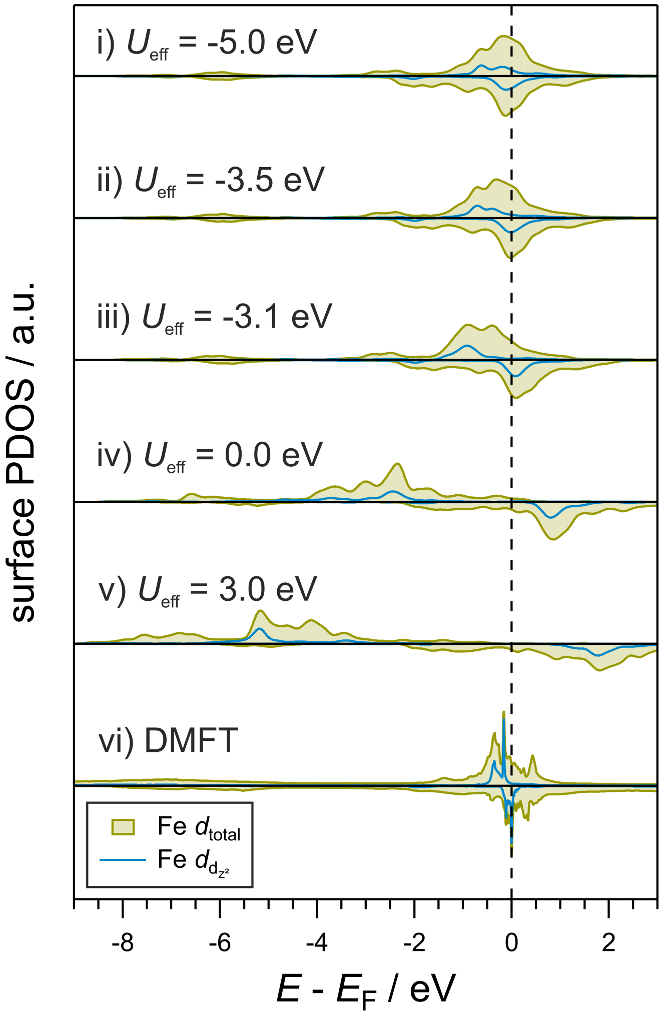

Figure S1: *Projected density of states (DOS) for the Fe(100)-p(1 × 1)O surface, comparing different theoretical approaches. The plots illustrate the evolution of the electronic structure for different settings of U_{eff} in the DFT+U formalism (i-v), juxtaposed to the DOS obtained from optimized DMFT calculations (vi). Notably, the curve presented in iv) does not strictly stem from DFT+U calculations but rather uncorrected DFT calculations, where no U_{eff} is applied. The results displayed in vi) stem from DMFT calculations that utilize optimized values of U = 3.1 eV and J = 0.8 eV (note the positive signs). This comparison illustrates how negative values of U_{eff} can approximate the DMFT results by reducing the d-band exchange splitting. However, the DFT+U approach fails to capture many-body effects, particularly evident in the oxygen bands between -5 eV and -9 eV, which in DMFT are broadened due to spin-dependent lifetime effects and interaction with many-body satellites, features absent in DFT+U simulations.*

S2: Momentum map simulation

The theoretical momentum maps shown in Fig. 2d of the main manuscript were generated from gas-phase DFT calculations of the pentacene (5A) highest occupied molecular orbital (HOMO). The momentum distribution was computed as the squared modulus of the orbital's Fourier transform, evaluated on an isoenergetic sphere corresponding to the kinetic energy of the photoelectrons (35 eV).[2] This yields the base momentum map shown in Fig. S2a.

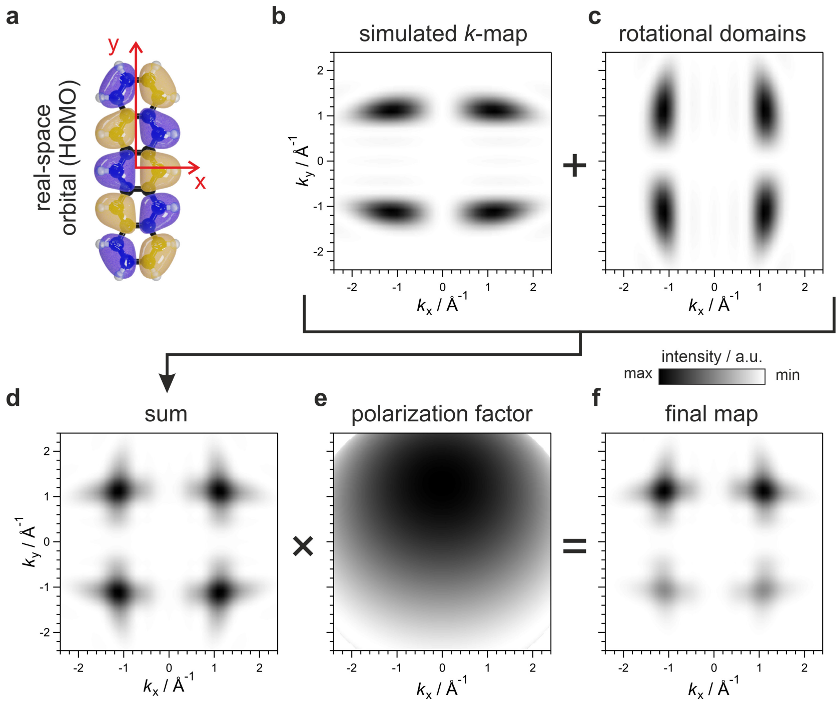

Figure S2: *Workflow for generating theoretical momentum maps of pentacene.* ***a)*** *Real-space representation of the HOMO of 5A, obtained from gas-phase DFT calculations.* ***b)*** *Corresponding momentum map extracted from the Fourier transform of the orbital.* ***c)*** *Simulated map for molecules arranged in rotationally equivalent domains.* ***d)*** *Sum of the individual domain contributions, accounting for spatial averaging inherent in ARPES measurements.* ***e)*** *Polarization factor that reflects the experimental light incidence and polarization geometry.* ***f)*** *Final theoretical momentum map resulting from the multiplication of the summed domain map with the polarization factor. This final image serves as the theoretical counterpart to the experimentally measured photoemission intensity distributions.*

Because angle-resolved photoemission spectroscopy (ARPES) probes large lateral areas, it effectively averages over different molecular orientations on the surface. On the Fe–O substrate, the presence of azimuthally rotated molecular domains must be taken into account. The substrate's fourfold symmetry allows for a simple rotational treatment: a 90° rotation of the base map (Fig. S2b) yields the equivalent domain shown in Fig. S2c. Since additional symmetry operations would only reproduce features already present in these maps, we limit domain averaging to these two rotationally equivalent components. Their sum (Fig. S2d) approximates the domain-averaged momentum distribution expected in the experiment. Finally, a polarization factor, based on the photon energy and incidence angle used in the experiment, is applied to account for matrix element effects, resulting in the final theoretical momentum map shown in Fig. S2e.

S3: Additional ARPES data of the Fe-O/5A interface

To identify the molecular peaks, we examine the momentum-resolved photoemission maps before and after 5A adsorption. Figure S3 shows intensity maps recorded at approximately -1.5 eV (relative to E_F) for a) the clean Fe–O surface, b) 5A adsorbed on Fe–O, and c) the calculated gas-phase HOMO of 5A. For the map associated with the HOMO, which we identify based on the gas-phase simulation (panel c), we find that the measured distribution after 5A adsorption (panel b) exhibits a strong resemblance to both the gas-phase orbital and the pre-existing features of the clean substrate (panel a).

Notably, the agreement between experimental momentum maps and gas-phase orbital calculations is a commonly observed behavior in photoemission tomography, even in systems with significant hybridization.[3] This can be attributed to the robustness of the orbital nodal structure, which remains largely unaffected even by the formation of bonding and antibonding states during chemisorption.

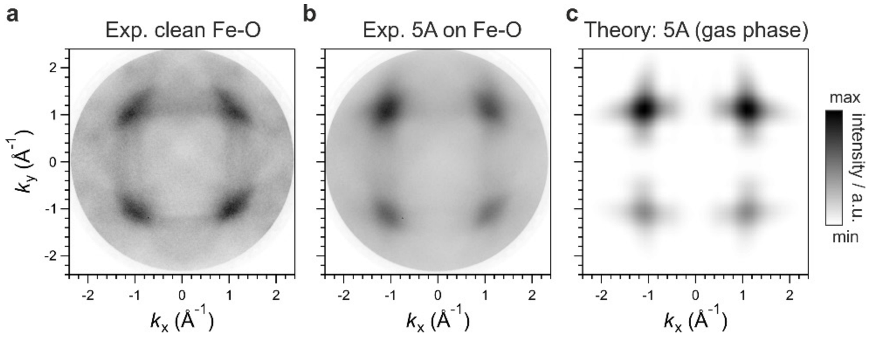

Figure S3: *Momentum-resolved photoemission intensity maps ($E - E_F \approx$ **-1.5** eV) comparing* ***a)*** *the clean Fe–O substrate,* ***b)*** *5A adsorbed on Fe–O, and* ***c)*** *the gas-phase HOMO of 5A from theory.*

To further evaluate the impact of pentacene adsorption on the electronic properties of the Fe-O substrate, we analyzed the momentum-resolved data and surface band structure, as shown in Fig. S4. Notably, in the energy region near the Fermi level, no significant alterations in the surface electronic structure or band structure of Fe-O are observed. This is evident in the momentum-resolved photoemission maps presented in Fig. S4a and Fig. S4c, corresponding to Fe-O and 5A/Fe-O, respectively, as well as the surface band structures shown in Fig. S4b and Fig. S4d.

This indicates that 5A does not induce substantial modifications to the substrate's electronic structure or its band structure. Furthermore, the absence of detectable charge transfer in our data supports this conclusion, as it implies that the *d*-shell occupation remains unchanged, thereby preserving the substrate's many-body scattering properties.

To further corroborate that the substrate's electronic structure is unaffected by the adsorbate, we present momentum maps and band structure cuts before and after 5A adsorption. While new resonances emerge at approximately -1.6 eV and -2.8 eV following 5A adsorption, no changes are observed in the energy region close to the Fermi level, specifically within the [0.0, 0.5] eV range.

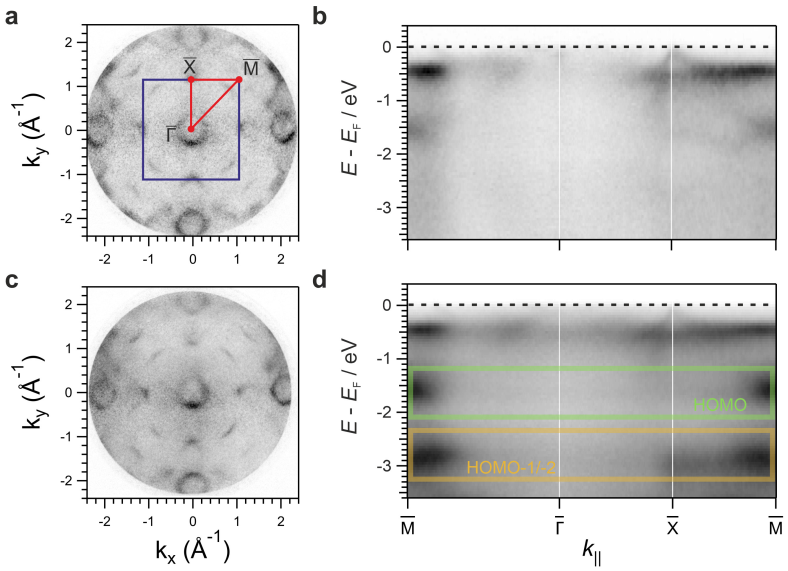

Figure S4: *Momentum-resolved data of clean Fe-O and 1 ML 5A on Fe-O.* ***a),c)*** *Momentum maps at the Fermi energy are shown for* ***a)*** *bare Fe-O and* ***c)*** *1 ML 5A on Fe-O. The blue square in a) marks the 1st surface Brillouin zone of the Fe-O surface, and red lines indicate the corresponding high-symmetry directions. By slicing the entire 3D data cube along these high-symmetry lines, the surface band structure is derived.* ***b),d)*** *Resulting surface band structures of the* ***b)*** *Fe-O substrate and the* ***d)*** *5A/Fe-O system. For clarity, the two molecular peaks discussed in the main text (see Fig. 2) are highlighted by colored boxes. Data were acquired using p-polarized light at a photon energy of 40 eV.*

S4: Characterizing the molecule-induced valence band peaks

In this study, we present a detailed analysis of the valence band peaks corresponding to the highest occupied molecular orbital (HOMO) and its adjacent orbitals, HOMO-1 and HOMO-2. Our approach employs a fitting model using a sum of Gaussian functions combined with a linear background. While this method offers a straightforward means of modeling the peaks, the underlying complexity of the peak structure may not be fully captured. As such, the precise number of peaks contributing to these broad features remains uncertain. However, by assessing the goodness-of-fit for various fitting functions, we can infer the minimum number of peaks potentially present in the spectra.

To quantify the error in our measurements, we first fit a flat region of the spectrum with a linear function, as illustrated in Fig. S5a-b. The residuals from this fit allow us to estimate the noise amplitude in our measurements as the standard deviation, yielding a value of 3.6 (in arbitrary units). Given that our analysis involves the subtraction of two spectra, the error, calculated according to standard Gaussian error propagation methods, is effectively multiplied by a factor of $\sqrt{2}$. This assumption is reasonable, considering the error is likely consistent across both measurements.

We then conducted a series of fits for the HOMO peak using one, two, and three Gaussian functions, as shown in Fig. S6a-c. The residuals clearly indicate that a single Gaussian is insufficient to accurately reproduce the experimental data, exhibiting significant and non-uniform intensity variations. To quantitatively evaluate the fit quality, we calculated the dimensionless goodness-of-fit parameter χ^2,[4] defined as:

$$\chi^2 = \sum_{i=1}^{N} \left(\frac{y_i - y(x_i)}{\alpha_i} \right)^2 ,$$

where y_i represents the measured values, $y(x_i)$ denotes the fitted values, and $\alpha_i = \alpha = 5.1$ corresponds to the estimated error from Fig. S5. The fit quality of each fit is then assessed by the quantity

$$\chi_\nu^2 = \frac{\chi^2}{\nu},$$

which takes into account the number of degrees of freedom ν of each fit. The value of ν is obtained from the difference $\nu = N - N_{\text{cons.}}$, where $N_{\text{cons.}}$ corresponds to the number of constraints, *i.e.* the number of fit parameters, and N to the total number of data points.

The resulting χ_ν^2 values for the different fits were $\chi^2_{\nu,\text{HOMOa}} = 7.3$ for the single Gaussian fit, and $\chi^2_{\nu,\text{HOMOb}} = 1.3$ and $\chi^2_{\nu,\text{HOMOc}} = 1.0$ for the two- and three-Gaussian fits, respectively. Typically, a χ_ν^2 value close to or below 1 is indicative of a good fit. Our analysis reveals that the single Gaussian model significantly underperforms, as evidenced by both the residuals and the elevated χ_ν^2 value.

For the HOMO-1/-2 peaks, a similar fitting procedure was applied, as depicted in Fig. S7. Due to only marginal improvements in fit quality, we limited the analysis to a single Gaussian and a sum of two Gaussians, yielding values of $\chi^2_{\nu,\text{HOMO}-1/-2\text{a}} = 1.6$ and $\chi^2_{\nu,\text{HOMO}-1/-2\text{b}} = 1.4$. Additionally, we explored fits using Lorentzian functions instead of Gaussians; however, the Lorentzian fits consistently produced inferior results, as indicated by higher χ^2 values.

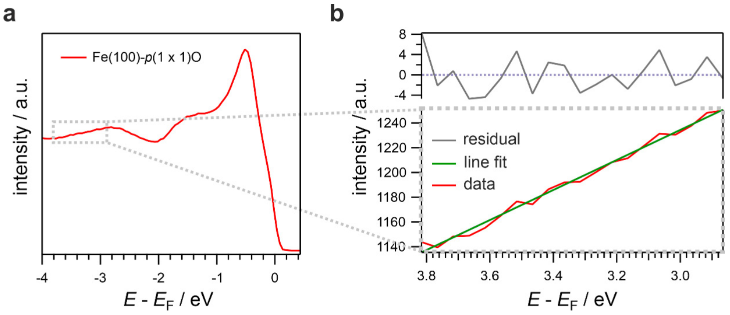

Figure S5: *Determination of the noise level in the photoemission data.* ***a)*** *Momentum-integrated valence band spectrum of a freshly prepared Fe-O surface. The dotted grey box indicates a region that exhibits approximately linear behavior.* ***b)*** *Close-up of the linear region (red curve) with a corresponding linear fit (green line). The inset at the top shows the residuals of this fit (grey curve), with the mean value of the residuals highlighted by a dotted violet line. The standard deviation of the residuals is considered the error margin for a single photoemission experiment.*

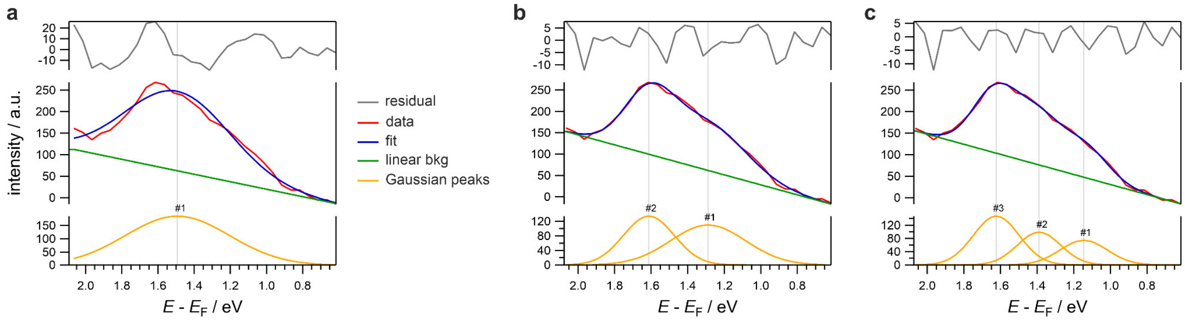

Figure S6: *Fitting analysis of the HOMO-related photoemission peak for the 5A/Fe-O interface, using a linear background combined with varying numbers of Gaussian components.* ***a)*** *Fit with a single Gaussian. The residuals (grey curve) show significant amplitude and systematic structure, indicating that this model is inadequate for accurately describing the HOMO peak.* ***b)*** *Fit with two Gaussians.* ***c)*** *Fit with three independent Gaussians. The progression from a single to multiple Gaussians improves the fit, suggesting the complexity of the HOMO feature at the 5A/Fe-O interface.*

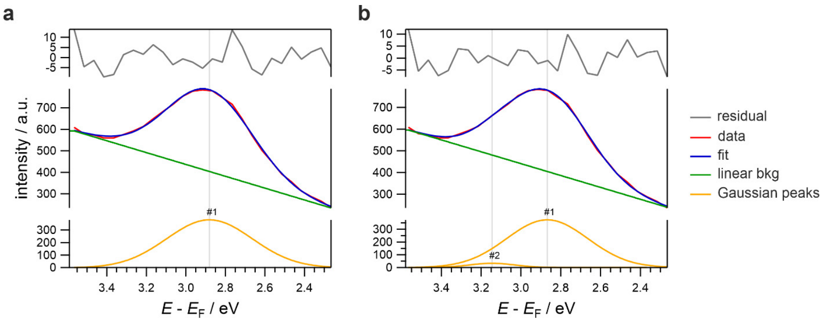

Figure S7: *Fitting analysis of the HOMO-1/-2-related photoemission peak for the 5A/Fe-O interface.* ***a)*** *Fit using a fitting function consisting of a single Gaussian combined with a linear background.* ***b)*** *Fit results for a sum of two Gaussians and a linear background.*

S5: Properties of 5A in the sub monolayer regime

In the main text, we focused on the structural characterization of the saturated monolayer (1 ML) film of 5A molecules adsorbed on the Fe-O substrate. Here, we present the results of control experiments conducted on a 5A film with half the coverage, *i.e.*, 0.5 ML.

For the 0.5 ML film, unlike the 1 ML film, no distinct diffraction pattern was observed in Low-Energy Electron Diffraction (LEED) experiments. However, large-scale Scanning Tunneling Microscopy (STM) images reveal the onset of self-assembly into ordered islands (Fig. S8a). A close-up examination of these islands indicates a (5, 4; 5, -4) molecular unit cell, which corresponds to roughly half the molecular density compared to the structure identified for the 1 ML film.

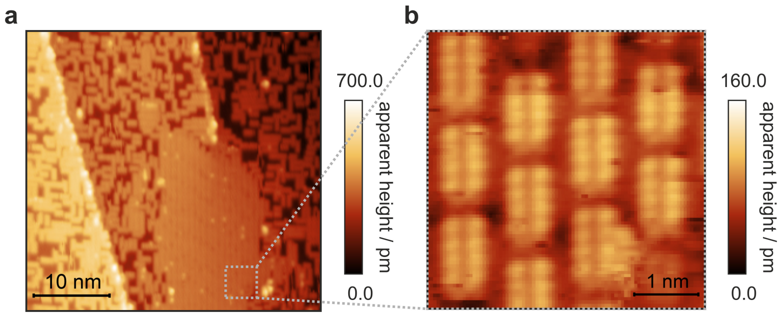

Figure S8: *STM images of a 0.5 ML film of 5A adsorbed on Fe-O.* ***a)*** *Large-scale STM image obtained at -2.0 V bias voltage and 20 pA tunneling current, showing an ordered island of 5A molecules.* ***b)*** *Close-up image of an ordered island, revealing a uniform molecular arrangement that resembles a (5, 4; 5, -4) superstructure. The image was recorded at -2.5 V bias voltage for a tunneling current of 100 pA.*

To investigate whether this difference in molecular assembly impacts the electronic structure, Scanning Tunneling Spectroscopy (STS) measurements were also performed on the 0.5 ML film. The resulting spectrum, shown as the light blue curve in Fig. S9a, is compared to the spectrum of the 1 ML film (dark blue curve). Further analysis using differential conductance (dI/dV) maps, recorded at bias voltages of -2.0 V and +2.0 V (Fig. S9c), reveals a notable resemblance to the gas-phase HOMO and LUMO of 5A (Fig. S9b). This similarity confirms that the distinctive three-peak structure observed in the unoccupied electronic states, as probed by STS, originates from the LUMO.

To further elucidate the electronic structure, we performed additional photoemission experiments on the 0.5 ML film (Fig. S10a). These experiments display the momentum-integrated valence band spectra of both the Fe-O substrate and the 5A-covered Fe-O interface. Although these measurements, obtained using a lab-based light source, are inherently noisier, the peak structures align well with those from the 1 ML film.

The observed reduction in work function, which is reduced by 0.22 eV for the 0.5 ML film and by 0.36 eV for the 1 ML film, is consistent with the lower molecular density of the 0.5 ML film. This reduction is primarily attributed to the push-back effect caused by Pauli repulsion, as there is no significant charge transfer between the 5A molecules and the Fe-O substrate.[5]

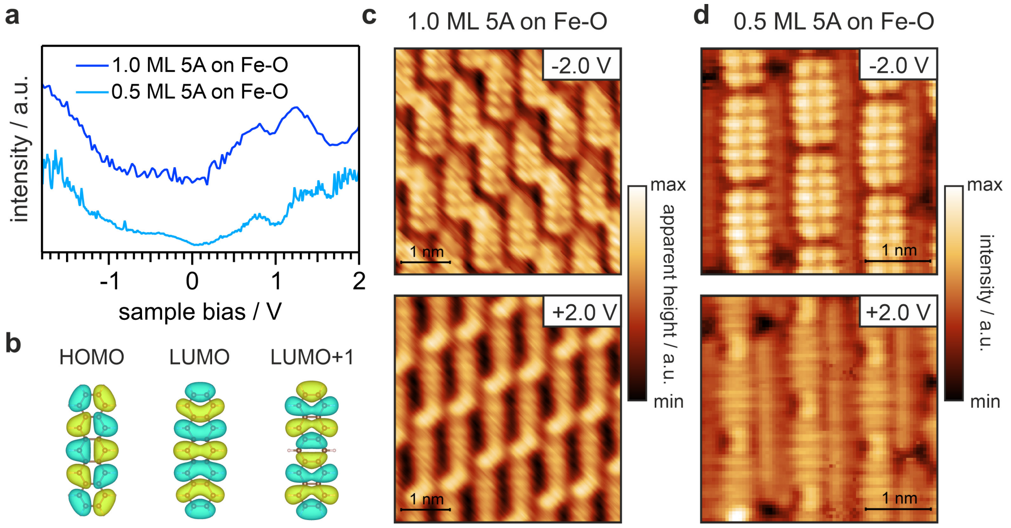

Figure S9: *Comparison of STM and STS results for a 1 ML versus a 0.5 ML film of 5A molecules on Fe-O.* ***a)*** *Plots of the measured STS spectra for the 0.5 ML (light blue curve) and the 1 ML 5A (dark blue curve) films. Both curves give rise to very similar spectroscopic characteristics, including three peaks at 0.8 V, 1.2 V, and 2.0 V in the region of the unoccupied states, as well as one broad resonance centered at approximately -1.6 V. In addition, we find some intensity located near -0.5 eV, however, due to the absence of clear molecular features, we attribute this intensity to the high substrate DOS in this region.* ***b)*** *Orbital plots depicting the gas phase HOMO, LUMO, and LUMO+1 of 5A.* ***c)*** *STM images of a 1 ML film of 5A on Fe-O, obtained at -2.0 V (top) and +2.0 V (bottom). The top image was recorded with a tunneling current of 500 pA, while the bottom image was captured for a tunneling current of 100 pA.* ***d)*** *dI/dV maps of an ordered island of a 0.5 ML film of 5A on Fe-O. The top image corresponds to a bias voltage of -2.0 V, and the bottom image to a bias voltage of +2.0 V. Both maps were recorded for a tunneling current of 300 pA. Comparison of the experimentally obtained images shows that in both cases — whether at sub-monolayer coverage or at full monolayer coverage — the electronic states in the bias voltage ranges of [-2.0, -1.0] V and [0.0, 2.0] V are clearly associated with the HOMO and LUMO of 5A, respectively.*

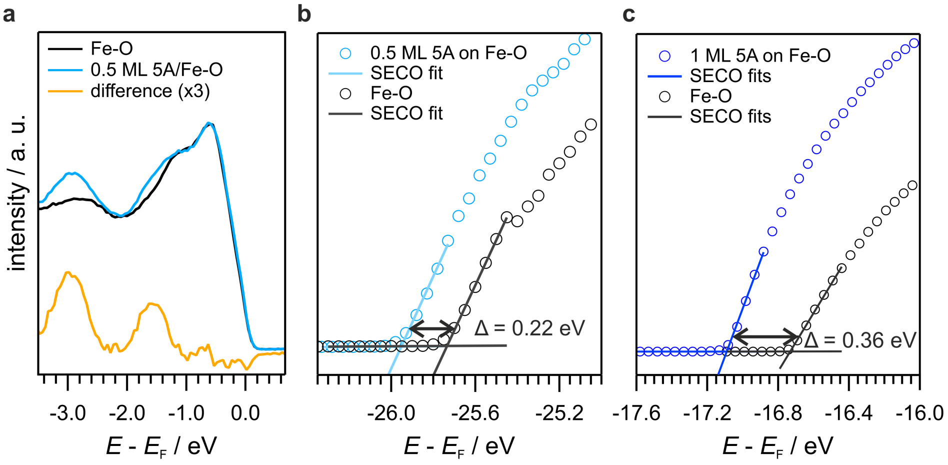

Figure S10: *Photoemission data for 0.5 ML and 1 ML of 5A adsorbed on Fe-O.* ***a)*** *Momentum-integrated valence band spectra of the clean Fe-O substrate (black curve) and the 0.5 ML 5A/Fe-O system (light blue curve).* ***b),c)*** *Evaluation of the work function shift by analyzing the secondary electron cut-off before and after 5A deposition for the 0.5 ML and 1 ML systems, respectively. The work function of the clean Fe-O substrate is $\Phi_{Fe-O} = (4.44 \pm 0.06\,)\,eV$, derived from an average of seven independently prepared samples. The error corresponds to the mean average error. Upon 5A deposition, the work function decreases by 0.22 eV for 0.5 ML coverage, and by a total of 0.36 eV for 1 ML coverage. This reduction, in the absence of effective charge transfer, can be attributed primarily to the push-back effect due to Pauli repulsion. Data shown in a) and b) were recorded using an HHG-based light source at a photon energy of 29.7 eV, providing p-polarized light, while the data in c) were acquired using an unpolarized He discharge lamp at a photon energy of 21.2 eV.*

S6: PDOS of a free-standing pentacene layer

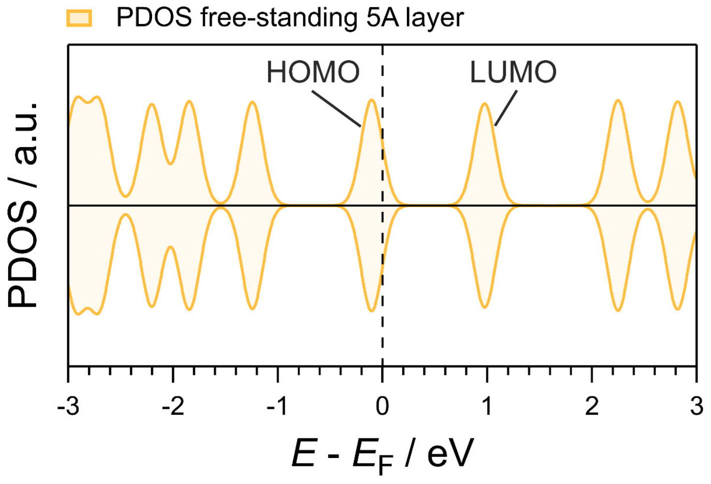

Figure S11: *PDOS for a free-standing layer of 5A. The arrangement of the molecules matches the one of the identified (3, 1; -1, -6) superstructure for the saturated monolayer (1 ML) on top of Fe-O.*

S7: Line profiles of the LUMO states

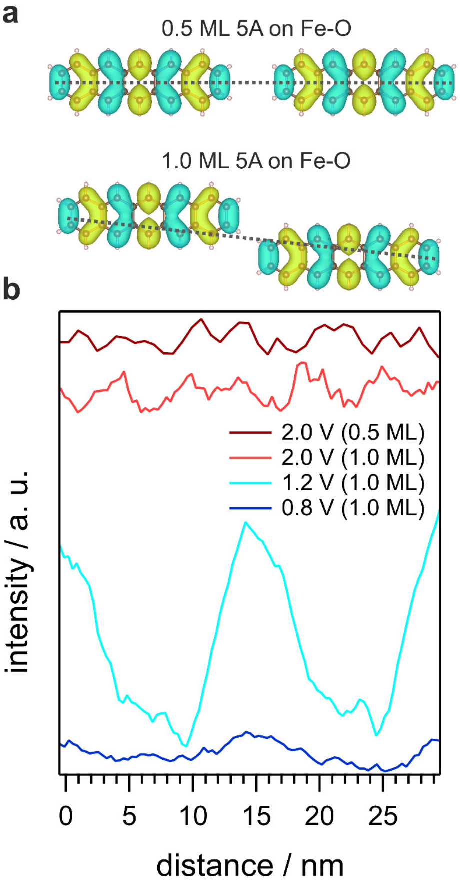

Figure S12: *Line profiles of the LUMO-related dI/dV maps.* ***a)*** *Sketches showing how the line profiles are extracted for measurements on the 0.5 ML (top) and the 1 ML film (bottom) of 5A on Fe-O.* ***b)*** *Corresponding line profiles of the identified LUMO features extracted from the STM images.*

S8: Simulating STM features from the calculated LUMO densities

To further validate the experimental findings, we calculated the integrated electron densities for the three LUMO peak positions that were identified in Fig. 3. These simulations provide insight into the spatial distribution of the molecular orbitals contributing to the measured d*I*/d*V* signal.

By applying a cutting plane at the estimated tip-molecule distance, we obtain the same characteristic features for the LUMO like the ones observed in the constant-height STM simulations, underscoring the robustness of our orbital assignments.

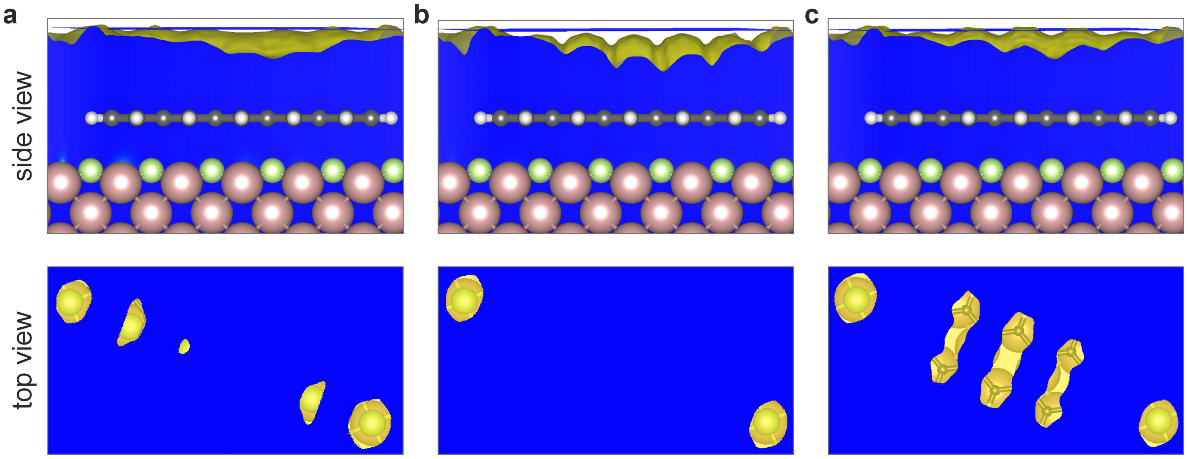

Figure S13: *Integrated density plots for the energy regions corresponding to the identified LUMO features. Panels* ***a)***, ***b)***, *and* ***c)*** *depict density simulations near +0.1 V, + 0.5 V, and +1.2 V, equivalent to the STM energy windows highlighted in Fig. 3d, respectively. The top row shows side views of the calculated densities, with the thin blue lines indicating the cutting plane at the estimated tip-molecule distance. The bottom row presents top-view images of the electron densities above this cutting plane, offering a direct comparison to the dI/dV images in Fig. 3. For clarity, the atomic positions of the molecule and the surface were superimposed on the density plots. Iso-value thresholds were optimized to best align with the STM-equivalent simulations, set at* 6.0×10^{-8}, 1.2×10^{-7}, *and* 2.5×10^{-8} *for a), b), and c), respectively.*

S9: Changes in the 5A MOPDOS for different values of *U* and *J* resulting from DFT+U

As previously discussed, the DFT+U method effectively captures the correlation-induced modifications of the d-bands. Here, we explore the impact of these modifications on the interaction between the substrate and subsequently adsorbed pentacene (5A) molecules.

In Fig. S14, we present a summary of the molecular projected density of states (MOPDOS) for the 5A/Fe-O interface, corresponding to different U_{eff} settings. As U_{eff} becomes more negative (from top to bottom), the exchange splitting reduces and the frontier orbitals of 5A exhibit increasingly pronounced signs of chemical interaction with the substrate. This observation aligns well with recent findings by Cao et al.,[6] which demonstrate that a reduction in the exchange splitting of the *d*-band enhances the chemical reactivity of ferromagnetic transition metal surfaces. Consequently, the tuning of the U_{eff} parameter in DFT+U not only affects the electronic structure of the substrate but also plays a critical role in determining the nature and strength of molecule-substrate interactions at the 5A/Fe-O interface.

Notably, while the HOMO does only split in two well-defined states, even at large negative U_{eff} values, a much more textured peak structure is found for the LUMO. In accordance with the main text, we attribute this behavior to the non-uniform overlap of the LUMO lobes with the subjacent Fe atoms.

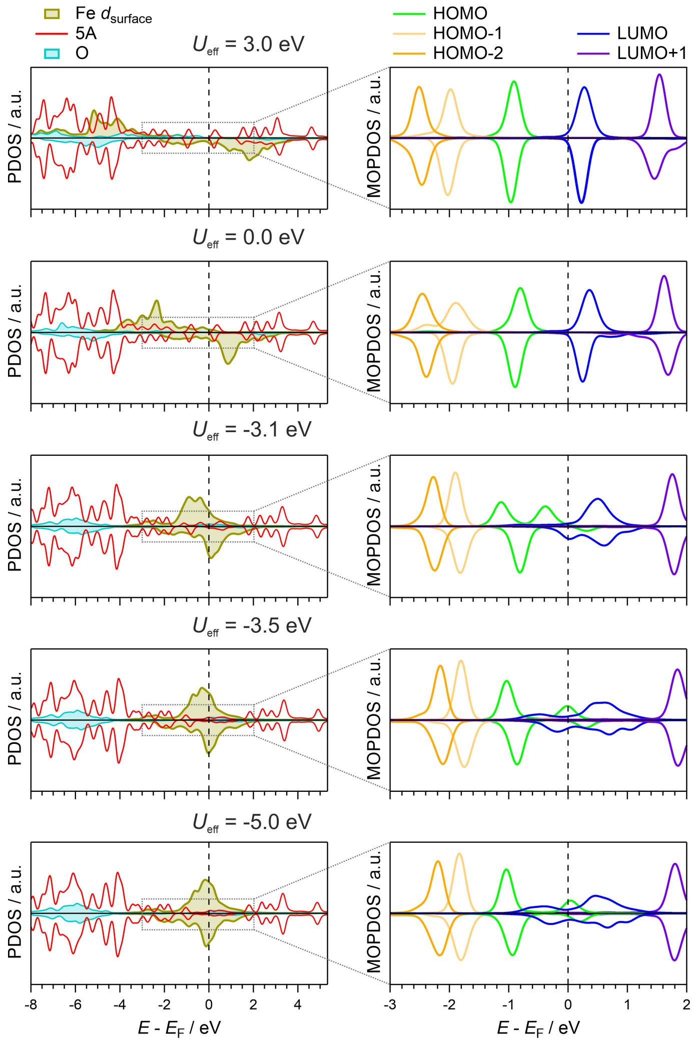

Figure S14: *Impact of U and J settings on the DOS of Fe-O and MOPDOS of pentacene (5A). Molecular projected density of states (MOPDOS) for the 5A/Fe-O interface, presented alongside the substrate's d-PDOS for various* U_{eff} *settings in the DFT+U method. As* U_{eff} *becomes more negative, reducing the exchange splitting, the frontier orbitals of 5A show increasingly strong chemical interactions with the substrate. This trend underscores the critical role of* U_{eff} *in influencing both the substrate's electronic structure and the molecule-substrate interactions at the 5A/Fe-O interface.*

S10: Chemisorption energy of atomic nitrogen for different U_{eff} parameters

In our manuscript, we specifically examine the interaction between the Fe-O surface and adsorbed 5A molecules. To explore whether the principles we deduced could play a decisive role in catalytic processes, we conducted model simulations in which 5A was replaced with a more catalytically relevant species, atomic nitrogen (N), and analyzed the resulting interface.

Consistent with recent findings by Cao et al.,[6] we observed that reducing the exchange splitting leads to a significant increase in the chemisorption energy of nitrogen (see Fig. S15). However, unlike Cao et al., who achieved this effect by setting the spin to zero in all atoms, our approach involved simulating the influence of electron correlation, which naturally reduces magnetism.

Our results not only corroborate the concept of spin catalysis—where surface magnetism is pivotal in determining chemisorption energies—but also underscore the importance of many-body effects in surface reactivity. Properly accounting for these effects could provide deeper insights into the electro- and photocatalytic behavior of transition metal surfaces and the occurring reaction pathways.

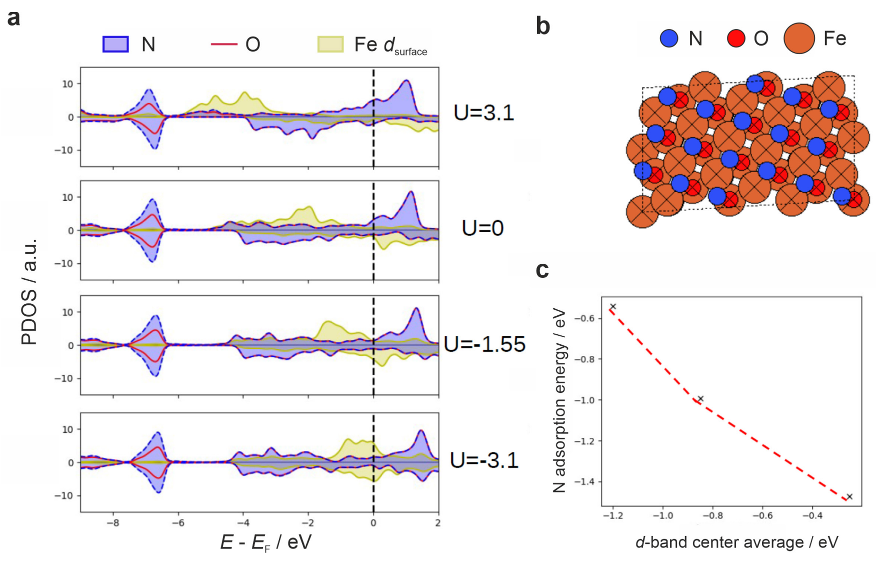

Figure S15: *Influence of exchange splitting on the electronic structure and adsorption properties of nitrogen on Fe-O.* ***a)*** *Projected density of states (PDOS) for nitrogen (N) adsorbed on the Fe-O surface, displaying the Fe d-bands and N states across four different U_{eff} parameter settings. The top curve corresponds to the highest U value, while the bottom curve represents the lowest (highly negative) U_{eff} value.* ***b)*** *Adsorption site for N on the Fe-O surface, determined through structural optimization.* ***c)*** *Dependence of the adsorption energy of N on the average surface d-band center, which shifts as the U_{eff} parameter is varied. The upward shift of the d-band center is accompanied by a reduction of the exchange splitting.*

S11: Influence of the adsorption site on the 5A-MOPDOS

To evaluate the impact of the adsorption site on the 5A/Fe-O interface, we compared the optimized 5A configuration (Fig. S16a) with an alternative configuration where the molecules are shifted by half a substrate unit cell vector along the oxygen rows, as shown in Fig. S16c. Due to this shift, the central carbon atoms are no longer positioned directly above the Fe surface atoms but lie between them.

For this new configuration, we performed additional DFT+U calculations using $U_{\mathrm{eff}} = -3.1$ eV, the same value applied in the DFT+U calculations presented in the main text. The resulting PDOS (Fig. S16d) indicates that the spin-dependent signatures of strong chemical interactions — specifically, the splitting and broadening of the frontier molecular orbital states (Fig. S16b) — almost entirely disappear for this modified adsorption configuration. Instead, the HOMO- and LUMO-related resonances emerge as single peaks in both spin channels. This behavior aligns with our model prediction that the extent of hybridization effects is critically dependent on the spatial overlap between the Fe *d*-states and the frontier molecular orbital lobes (see also Fig. 6 in the main text).

We further simulated STM images of the LUMO states for this shifted adsorption configuration that are summarized in Fig. S16e. Notably, these simulations exhibit patterns that strongly deviate from the experimental observations (see Fig. 3), illustrating that the adsorption site significantly influences the electronic structure and interaction of the 5A molecules with the Fe-O substrate.

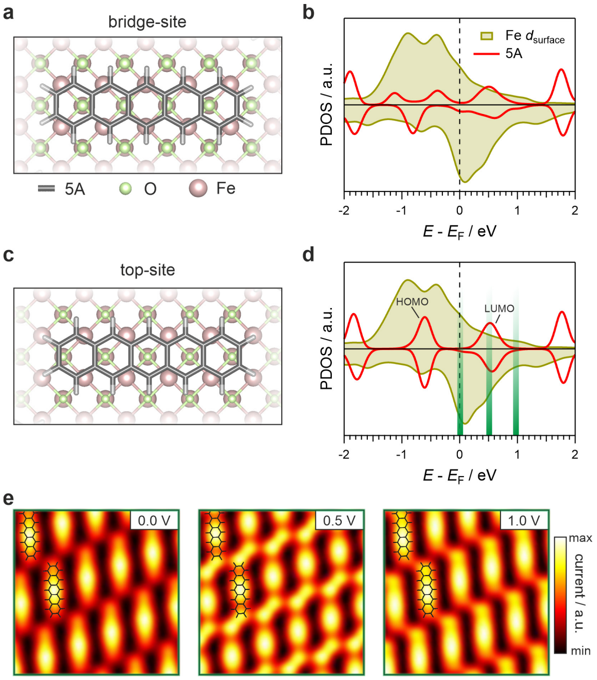

Figure S16: *Influence of Adsorption Geometry on the Electronic Structure of 5A.* ***a)*** *Optimized adsorption configuration for 5A molecules on the Fe-O surface and* ***b)*** *the resulting PDOS.* ***c)*** *Arrangement of a 5A molecule shifted by half a substrate unit cell vector along the oxygen rows relative to the optimized configuration.* ***d)*** *PDOS obtained for the adsorption geometry shown in (c).* ***e)*** *Simulated scanning tunneling microscopy (STM) images for selected energy regions highlighted in (d). These STM patterns do not align with experimental observations, highlighting the inadequacy of the shifted adsorption geometry in replicating the electronic properties and surface interactions of 5A. The results emphasize the critical role of the adsorption site in determining the electronic structure and corresponding STM features. All data were obtained using DFT+U with* $U_{eff} = -3.1$ *eV.*

S12: Determining the slopes of the lobe-functions in the *d*-band model

As explained in the main manuscript, we rationalize the splitting of the molecular states of 5A on passivated Fe in terms of the *d*-band model. For this, we have estimated the coupling strength, $V_{\mathrm{C-Fe}}$, between a C-atom state and a Fe-atom state from literature values of similar systems,[7–9] for the case that C is directly on top of Fe. While the standard *d*-band model predicts a splitting of the molecular states due to interactions with the Fe *d*-bands, as shown in Fig. 5 of the main manuscript, it fails to fully capture the behavior of the LUMO. For this reason, we aim to refine the *d*-band model — originally designed for small molecules on surfaces — and adapt it for larger adsorbates, as illustrated in Figure 6 of the main manuscript. In essence, we do not choose a uniform coupling strength for the whole molecule but assume a varying coupling along its long molecular axis. Thereby, we may explain the difference in behavior between the HOMO and LUMO level splitting with the different spatial distribution of the frontier orbitals, *i.e.* the shape of their lobes, and their relative position to the Fe atoms of the surface below. In the following, we want to demonstrate this influence of the orbital distribution on the *d*-band model, namely on $V_{\mathrm{C-Fe}}$, at the hand of an exemplary model calculation.

In our model, we divide the LUMO of 5A along its long axis according to its nodal structure into four separate regions (V1 - V4), as illustrated in Fig. S17 (the regions are doubled by symmetry). As the frontier orbitals of 5A are dominated by π-bonding character, *i.e.* with a nodal plane in the molecular plane, we then approximate each region by a single p_z orbital. The assumed p_z orbital is positioned such that the center of the density of the p_z orbital coincides with the center of the density of the respective LUMO region (marked by a yellow circle in Figure S17). In the next step, we assume that a major contribution to the molecule-surface interaction will consist of π-type bonding between the LUMO and Fe *d*-states. For this bonding, the d_{z^2} orbital is the one *d*-orbital, which symmetry matches best. Hence, for each region, we approximate the molecule-surface interaction as an interaction between a C(p_z) state, located at the center of the respective LUMO lobe, and a d_{z^2} orbital, located at the nearest Fe atom of the surface. In that way, we have reduced the complex LUMO distribution to several two-center problems, which we will now analyze separately. The resulting position vectors, $\vec{r}$, for the four regions are summarized in Table S1.

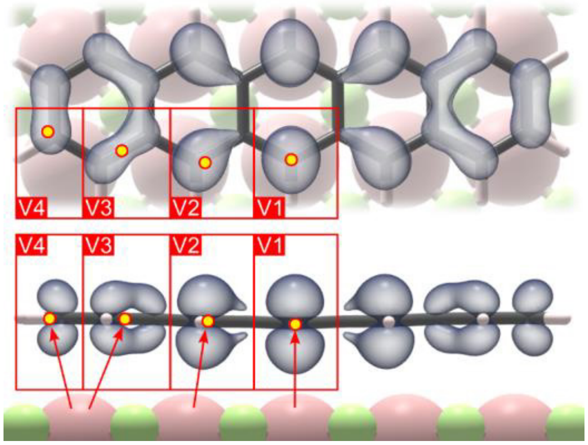

Figure S17: *Schematic of the four different coupling regions (V1-V4) representing different orbital lobes according to the nodal structure of the LUMO of 5A. The respective lobes are then approximated by* p_z *orbitals positioned at the yellow circles. The red arrows indicate the position vectors* $\vec{r}$.

Note, how the p_z-location changes from virtually on top a Fe atom in region V1 to a significant shift in region V3 and V4.

Table S1: *Distance, $|\vec{r}|$, and position vector $\vec{r}(x, y, z)$ of the C(p_z) orbitals that approximate the individual LUMO lobes relative to the respective Fe atom below. Values are given in Å.*

	V1	V2	V3	V4
x	-0.055	-0.128	-0.382	-0.872
y	0.024	0.557	1.290	-0.594
z	3.092	3.186	3.274	3.269
$\vert\vec{r}\vert$	3.093	3.237	3.539	3.435

Based on the stated approximations, the coupling strength for each LUMO region is given by Equation (1):

$$V_{\mathrm{C-Fe}} = \eta \frac{M_{\mathrm{C}} M_{\mathrm{Fe}}}{|\vec{r}|^{l_{\mathrm{C}}+l_{\mathrm{Fe}}+1}} \qquad (1)$$

where $|\vec{r}|$ is the *absolute* distance between the C and Fe atom in question and η is a structure factor containing the *relative* positioning of the two atoms.[7–9] For η, we refer to the Slater-Koster geometry factor for a σ-bond between a p_z and d_{z^2} state (see Eq. (2)), which is dependent on the directional cosine (l_x, m_y, n_z) of the respective position vector.[10] The resulting structure factors for each region are tabulated in Table S2.

$$\eta = n_z\left[n_z^{\,2} - \tfrac{1}{2}\left(l_x^{\,2} + m_y^{\,2}\right)\right] \text{ with } l_x = \cos\alpha_x\,, m_y = \cos\alpha_y\,, n_z = \cos\alpha_z \qquad (2)$$

Table S2: *Directional cosine (l_x, m_y, n_z) and structure factors, η, for a C(p_z)-Fe(d_{z^2}) sigma-bond.*

	V1	V2	V3	V4
l_x	-0.017	-0.040	-0.108	-0.254
m_y	0.007	0.172	0.364	-0.173
n_z	0.999	0.984	0.925	0.952
η	1.000	0.938	0.725	0.817

The coupling further depends on the angular quantum number l of the adsorbate state (p_z: l_C = 1) and the metal state (d_{z^2}: l_{Fe} = 2). The values M_C and M_{Fe} describe the potential around the atoms, however, these parameters are usually considered independent of the atomic positions.

As we want to illustrate the change of $V_{\mathrm{C-Fe}}$ with respect to the C-Fe position, we evaluate the coupling strength of each region relative to the case, where both atoms are directly on top of each other. The latter arrangement should show the largest coupling and correspond to the ideal case discussed in the main manuscript in Figure 5. This is effectively represented by region V1, which we, thus, take as our reference point.

For our purely positional analysis, the change in coupling strengths depends on the C-Fe separation as $\frac{1}{|\vec{r}|^4}$ as well as their relative position according to η. The changes in both parameters for regions V2-V4 in relation to V1 are compared in Table S3. Finally, we have to consider that the coupling strength enters the *d*-band model as V^2, or $\frac{1}{V^2}$ for the slope of the lobe-function, respectively. Therefore, we want to focus our discussion especially on the *overall* change in the coupling in the last row of Table S3. It is apparent that the coupling strength reduces significantly up to a factor of ≈ 5, when the assumed C-Fe distance increases. In other words, for regions V3 and V4, we expect a much steeper slope of the respective lobe-functions in the *d*-band model compared to region V1 or V2. This prediction is, indeed, in good agreement with the suggested coupling strengths in Fig. 6 of the main manuscript. Furthermore, even though we may consider the approximations rather crude, it demonstrates how the coupling of large adsorbates will change throughout the molecules. Consequently, the splitting of the respective molecular orbital may adopt a more complex structure than the usually expected two levels.

Table S3: *Relative reduction of the coupling strength due to C-Fe (Δr) separation and the structure factor ($\Delta\eta$) as well as overall influence on the lobe-functions (ΔV^2).*

	V1	V2	V3	V4
Δr	reference point	0.833	0.583	0.657
$\Delta\eta$		0.938	0.725	0.817
ΔV^2		0.611	0.178	0.288

S13: Additional d*I*/d*V* mapping for the HOMO

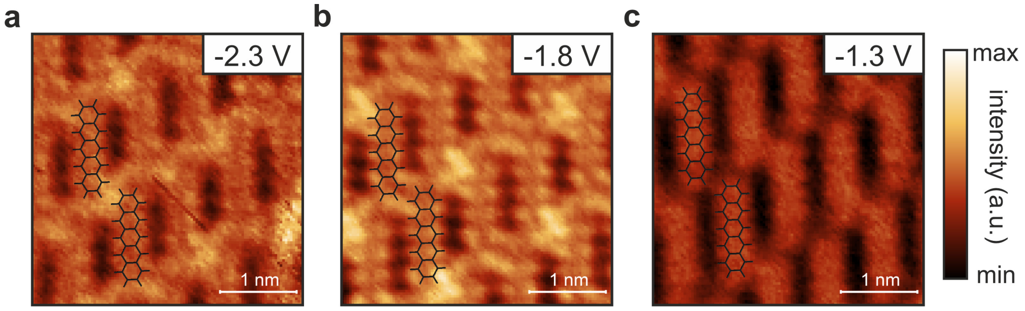

Figure S18: *Complementary dI/dV maps for the HOMO of 5A.* ***a)–c)*** *Maps recorded at bias voltages of -2.3 V, -1.8 V, and -1.3 V, respectively, with a tunneling current set to 300 pA for all images. Unlike the maps obtained for the LUMO energy window (see Fig. 3), the HOMO maps show no pronounced intensity variations across individual 5A molecules.*

In the main text, we presented a series of differential conductance (d*I*/d*V*) maps focused on the LUMO (see Fig. 3). Here, we provide complementary data by showing additional d*I*/d*V* maps that cover the energy region corresponding to the HOMO, ranging from [-2.3, -1.3] eV. These maps are displayed in Fig. S18.

Unlike the LUMO maps, the intensity patterns recorded for the HOMO do not exhibit pronounced variations across the 5A molecules. This observation is consistent with the fact that the HOMO is almost homogeneously distributed over the underlying Fe atoms. As a result, any variations due to molecule-substrate interactions are expected to be uniform within the surface plane.

However, it is worth noting that some notable intensity variations are observed at the points where the molecules are in close proximity to each other, particularly in Fig. S18b. Since our angle-resolved photoelectron spectroscopy (ARPES) data and theoretical simulations do not provide any evidence for intermolecular hybridization, we attribute these subtle intensity variations to a measurement artifact, evoked by the sign of the wave function between neighboring molecules.[11]

References:

1. Janas, D. M. *et al.* Enhancing Electron Correlation at a 3d Ferromagnetic Surface. *Adv. Mater.* **35**, 2205698 (2023).
2. Brandstetter, D., Yang, X., Lüftner, D., Tautz, F. S. & Puschnig, P. kMap.py: A Python program for simulation and data analysis in photoemission tomography. *Comput. Phys. Commun.* **263**, 107905 (2021).
3. Zamborlini, G. *et al.* Multi-orbital charge transfer at highly oriented organic/metal interfaces. *Nat. Commun.* **8**, 335 (2017).
4. Hughes, I. & Hase, T. *Measurements and their Uncertainties A practical guide to modern error analysis*. (Oxford University Press, 2010).
5. Hollerer, M. *et al.* Charge Transfer and Orbital Level Alignment at Inorganic/Organic Interfaces: The Role of Dielectric Interlayers. *ACS Nano* **11**, 6252–6260 (2017).
6. Cao, A. & Nørskov, J. K. Spin Effects in Chemisorption and Catalysis. *ACS Catal.* **13**, 3456–3462 (2023).
7. Nørskov, J. K. Effective medium potentials for molecule–surface interactions: H2 on Cu and Ni surfaces. *J. Chem. Phys.* **90**, 7461–7471 (1989).
8. Hammer, B. & Nørskov, J. K. Electronic factors determining the reactivity of metal surfaces. *Surf. Sci.* **343**, 211–220 (1995).
9. Vojvodic, A., Nørskov, J. K. & Abild-Pedersen, F. Electronic Structure Effects in Transition Metal Surface Chemistry. *Top. Catal.* **57**, 25–32 (2014).
10. Slater, J. C. & Koster, G. F. Simplified LCAO method for the periodic potential problem. *Phys. Rev.* **94**, 1498–1524 (1954).
11. Homberg, J., Gruber, M., Weismann, A. & Berndt, R. Enhanced conductance of molecular states at interstitial sites. *New J. Phys.* **25**, 013029 (2023).
